# Distinct nucleic acid interaction properties of HIV-1 nucleocapsid protein precursor NCp15 explain reduced viral infectivity

**DOI:** 10.1093/nar/gku335

**Published:** 2014-05-09

**Authors:** Wei Wang, Nada Naiyer, Mithun Mitra, Jialin Li, Mark C. Williams, Ioulia Rouzina, Robert J. Gorelick, Zhengrong Wu, Karin Musier-Forsyth

**Affiliations:** 1 Department of Chemistry and Biochemistry, Center for Retrovirus Research and Center for RNA Biology, The Ohio State University, Columbus, OH 43210, USA; 2 Department of Physics, Northeastern University, Boston, MA 02115, USA; 3 Department of Biochemistry, Molecular Biology and Biophysics, University of Minnesota, Minneapolis, MN 55455, USA; 4 AIDS and Cancer Virus Program, Leidos Biomedical Research, Inc., Frederick National Laboratory for Cancer Research, Frederick, MD 21702, USA

## Abstract

During human immunodeficiency virus type 1 (HIV-1) maturation, three different forms of nucleocapsid (NC) protein—NCp15 (p9 + p6), NCp9 (p7 + SP2) and NCp7—appear successively. A mutant virus expressing NCp15 shows greatly reduced infectivity. Mature NCp7 is a chaperone protein that facilitates remodeling of nucleic acids (NAs) during reverse transcription. To understand the strict requirement for NCp15 processing, we compared the chaperone function of the three forms of NC. NCp15 anneals tRNA to the primer-binding site at a similar rate as NCp7, whereas NCp9 is the most efficient annealing protein. Assays to measure NA destabilization show a similar trend. Dynamic light scattering studies reveal that NCp15 forms much smaller aggregates relative to those formed by NCp7 and NCp9. Nuclear magnetic resonance studies suggest that the acidic p6 domain of HIV-1 NCp15 folds back and interacts with the basic zinc fingers. Neutralizing the acidic residues in p6 improves the annealing and aggregation activity of NCp15 to the level of NCp9 and increases the protein–NA aggregate size. Slower NCp15 dissociation kinetics is observed by single-molecule DNA stretching, consistent with the formation of electrostatic inter-protein contacts, which likely contribute to the distinct aggregate morphology, irregular HIV-1 core formation and non-infectious virus.

## INTRODUCTION

**Figure 1.**
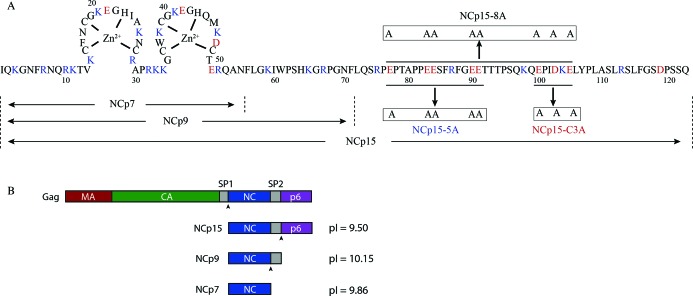
The HIV-1 NC proteins used in this study. (**A**) Sequence and structural features of NCp7, NCp9, NCp15 and the three NCp15 mutants (8A, 5A and C3A). Basic residues are in blue, acidic residues are in red and the ZFs are shown. (**B**) Three different forms of NC appear during HIV-1 maturation. The arrow heads indicate the cleavage sites that generate NCp15, NCp9 and NCp7.

Human immunodeficiency virus type 1 (HIV-1) nucleocapsid protein (NC or NCp7) is a small, 55-residue protein that contains two C-C-H-C-type zinc fingers (ZFs) and a basic N-terminal domain (NTD) (1) (Figure 1A). It plays numerous critical roles in the HIV-1 life cycle, such as facilitating viral assembly (2–5), genomic ribonucleic acid (RNA) packaging (6–9), RNA dimerization (10–13), reverse transcription (14–17) and integration (18–23). NC is also a nucleic acid (NA) chaperone protein that destabilizes stable NA structures and refolds them to a thermodynamically more favored state (14,16,17,24,25). HIV-1 NC's chaperone function is essential during reverse transcription of the highly structured viral RNA genome (gRNA) (26). For example, as a domain of the HIV-1 Gag precursor, HIV-1 NC is involved in the placement of the tRNA^Lys3^ primer onto the gRNA primer binding site (PBS) (27–29). Mature NC also plays roles in minus- and plus-strand transfer (16,17,30). Mutation of intact NC greatly reduces viral infectivity (7,31,32) and thus NC is a potential target protein for the development of new antiretroviral agents (17,33–36).

NC is first synthesized as a domain of HIV-1 Gag. During viral maturation, HIV-1 protease cleaves Gag into matrix (MA), capsid (CA), NCp7, p6 and two small peptides, spacer peptides 1 and 2 [SP1 and SP2, also referred to as p2 and p1, respectively (37)]. The cleavage reactions occur stepwise (38–40) in a highly regulated manner (37,41). Three different forms of NC appear sequentially during this process (Figure 1B). HIV-1 protease first cleaves after SP1, generating a form of NC called NCp15, which consists of mature NCp7 linked to SP2 and p6. Further processing of NCp15 results in NCp9 (NCp7 + SP2), and finally cleavage between NCp7 and SP2 results in release of mature NCp7 (Figure 1B). NCp15 and NCp9 appear only transiently but their lifetime is specifically programmed (41). Additional cleavage sites within NCp7 have been proposed based on *in vitro* peptide cleavage experiments (42), but NCp15, NCp9 and NCp7 are the three major forms that have been observed by the numerous studies mentioned thus far on Gag and NC precursor cleavage processes. Although all three forms of NC have an overall positive charge (p*I* = 9.50–10.15), the C-terminal p6 domain of NCp15 contains eight acidic residues and has a p*I* of 4.50 (Figure 1A).

Previous studies showed that mutation of the protease cleavage sites proximal to the NC domain will affect HIV-1 infectivity. The correct processing of the cleavage site between SP2 and p6 is critical for the virus and virus containing NCp15 is non-infectious (43–46). However, there is some debate regarding the effect of mutating the NCp7-SP2 cleavage site (expressing NCp9); some studies reported that mutations at this site lead to non-infectious virus (44,47), while in other studies a virus with NCp9 retains some infectivity (43,45,46,48). The different mutations introduced to block the processing of this cleavage site generate HIV-1 variants that likely have variable effects on other critical viral replication processes (e.g., assembly, core condensation, reverse transcription, integration, etc.) In addition, the experiments were performed in the context of different HIV-1 strains, which has been reported to lead to different outcomes (44,49). Therefore, it is difficult to determine the effect of authentic NCp9 and NCp15 on HIV-1 replication using cell culture assays.

HIV-1 NCp15 bears some resemblance to HTLV-1 NC, which has both a basic NTD containing the two ZF motifs and an acidic C-terminal domain (CTD). A previous study showed that the acidic CTD of HTLV-1 NC negatively regulates its chaperone activity (50). Based on a fluorescence study, it was suggested that the CTD folds back and interacts with the ZF domain (51). Additional biochemical studies suggested that the basic NTD of HTLV-1 NC interacts with the CTD either intramolecularly or intermolecularly, in the absence or presence of NAs, respectively (50). Whether a similar interaction regulates the chaperone activity of HIV-1 NCp15 is unknown.

The p6 domain of Gag interacts with several cellular and viral proteins (52), such as human proteins Tsg101 (53,54) and AIP/ALIX (55,56), and the viral accessory protein Vpr (57–59). Tsg101 and AIP/ALIX play roles during HIV-1 budding (53,54,56). Vpr has several functions during HIV-1 infection and is required for efficient viral replication in non-dividing cells (60–62). A significant amount of Vpr is also packaged into virions. Interestingly, it has been shown that p6 is excluded from the capsid core after HIV-1 maturation, while NC and Vpr are both located inside the core structure (63,64). It is possible that NCp15 can interact with these cellular and viral factors through p6, and these interactions could affect NCp15's chaperone function *in vivo*. Alternatively, the trafficking of the reverse transcription complex after HIV-1 cell entry may be negatively impacted by p6 fusion to NCp7/SP2.

In this study, we used biochemical and biophysical approaches to characterize and compare the NA binding and chaperone properties of mature NCp7 to those of authentic precursor proteins NCp9 and NCp15 that are only transiently found in virions (41). We also carried out cell-based assays to probe the potential roles of Tsg101 and Vpr interactions on the negative effect of NCp15 on virus infectivity. Our study supports the role of inter-protein contacts and abnormal protein–NA aggregate formation in the defects observed in NCp15-containing HIV-1.

## MATERIALS AND METHODS

### Preparation of proteins and NAs

The HIV-1 NC proteins and proviral plasmids used in this study were based on the pNL4-3 sequence (GenBank accession number AF324493) (65). The mutations denoted in Figure 1 were introduced as follows (nt positions based on AF324493): E77A (nts 2148 and 2150 changed from a to c; also introduces a BstUI diagnostic restriction site); E83A and E48A (nts 2166, 2168, 2169 and 2671 changed from a to c; also introduces a BstUI diagnostic restriction site); E90A and E91A (nts 2689, 2690 and 2692 changed from a to c; also introduces a BstUI diagnostic restriction site); and E100A, D103A and E105A (nts 2219, 2226, 2228 and 2234 changed from a to c; also introduce BanI, PvuI and StyI diagnostic restriction sites). Additionally, the mutant pNL4-3-based proviral plasmid that maintains NC in its p15 form (a gift from Davit E. Ott, Frederick National Laboratory for Cancer Research) (43) was further mutated, changing PTAP to LIRL (Figure 1A, amino acid positions 78–81) and/or FRFG to SRSG (Figure 1A, amino acid positions 86–89) to potentially disrupt Tsg101 and Vpr interactions with p6, respectively (53,66). For the PTAP to LIRL mutant, nts 2153 and 2156 were changed from c to t, nt 2157 was changed from a to t, nt 2158 was changed from g to c, nt 2159 was changed from c to g and nt 2162 was changed from c to t. For the FRFG to SRSG mutation, nts 2177 and 2183 were changed from t to c. Mutations were introduced using conventional molecular biology methods and confirmed by sequence analysis.

Unlabeled recombinant NC proteins were purified using a modification of a previously-described protocol (19). Briefly, *Escherichia coli* (E. coli) BL21(DE3) pLysS was transformed with pET32a-based (EMD Millipore, Billerica, MA, USA) NC expression plasmids. The NC proteins were expressed as fusion proteins with an N-terminal thioredoxin (Trx) tag and a tobacco etch virus (TEV) protease cleavage site (ENLYFQG) between the Trx and NC regions that allowed for release of Trx and generation of the authentic NC proteins shown in Figure 1A. After induction with 1 mM isopropyl β-D-1-thiogalactopyranoside (IPTG) at room temperature overnight, cells were harvested and resuspended in 100-mM *N*-cyclohexyl-3-aminopropanesulfonic acid buffer, pH 10.4 at 4°C. Sonication was used to lyse the cells and 2-mercaptoethanol (BME) was added to 1% final concentration. The pH of the buffer was lowered to ∼2 with 20% trifluoroacetic acid (TFA). The cell lysate, clarified by (i) ultracentrifugation at 51k × *g* in a Beckman (Beckman Coulter, Inc., Brea, CA, USA) Type 45 Ti rotor for 15 min at 4°C and (ii) filtering the resulting supernatant through a 0.45-μm cellulose acetate filter (Corning Life Sciences, Tewksbury, MA, USA), was loaded onto a reverse phase high-performance liquid chromatography (rp-HPLC) C18 column (Varian Microsorb 300–10, now Agilent, Santa Clara, CA, USA) and eluted with an acetonitrile gradient (10–90%) containing 0.1% TFA. The fractions corresponding to the desired product were collected and lyophilized. The fusion proteins were cleaved with 5-μg TEV protease per 100-μg protein (67,68) in a buffer containing 20-mM Tris-HCl, pH 7.4, 200-mM NaCl, 10-mM zinc acetate, 5-mM sodium citrate and 5-mM BME. The final cleaved NC product was further purified by rp-HPLC using a C18 column as described above. The rp-HPLC fractions, chosen by matrix-assisted laser desorption/ionization–time of flight mass spectrometry (MALDI-TOF MS), were quantified by amino acid analysis and combined with 1.1 equivalent of zinc-acetate per ZF then lyophilized and stored at −80°C. Final purity was gauged by amino acid analysis, amino acid sequencing and MALDI-TOF MS, and was found to be >95% pure (impurities were mainly NC proteins with minor N-terminal truncations). Chemically-synthesized NCp7 obtained from New England Peptide (Gardner, MA, USA) was used in some studies. It was re-folded with 1.1 equivalents of zinc ions per ZF in a buffer containing 20-mM HEPES, pH 7.5 and 0.1-mM tris(2-carboxyethyl)phosphine (TCEP). The concentrations were determined by measuring the ultraviolet absorbance at 280 nm using the following extinction coefficients: NCp7: 5690 M^−1^ cm^−1^; NCp9: 11 380 M^−1^ cm^−1^; NCp15 and NCp15 mutants: 12 660 M^−1^ cm^−1^.

^15^N- and ^13^C-labeled HIV-1 NC proteins were prepared under native conditions using a modification of a previously-described method (69). Briefly, *E. coli* BL21(DE3) pLysS was transformed with pET32a-based NCp7 expression plasmid (expressing Trx-NCp7) or pET3a-based (EMD Millipore) NCp15 expression plasmids. Bacteria were grown in minimal media supplemented with ^15^N-NH_4_Cl and/or ^13^C-Glucose as sole nitrogen and carbon source and were induced with 1-mM IPTG at room temperature overnight. Bacteria were harvested and resuspended in a buffer containing 50 mM Tris-HCl, pH 7.4, 200-mM NaCl, 1-mM Zn(OAc)_2_, 10% glycerol and 10-mM BME (loading buffer). After lysis of the bacteria by sonication, polyethylenimine (PEI) was added to a final concentration of 0.5% to precipitate NAs. The Trx-NCp7 fusion protein was precipitated by 25% ammonium sulfate and cleaved by TEV protease as described above (67,68). After the TEV cleavage step (for NCp7) and after the PEI precipitation step (for NCp15), proteins were purified on a HiTrap SP Sepharose FF 5-ml column (GE Healthcare, Pataskala, OH, USA) using the above loading buffer and eluted with a linear NaCl gradient ranging from 200 mM to 1 M. The crude product from the SP column was further purified on a HiLoad Superdex 75 gel filtration column (GE Healthcare). The purified ^15^N- or ^15^N/^13^C-labeled NCp7 and NCp15 proteins were concentrated to ∼0.5 mM using Amicon spin concentrators and dialyzed against degassed nuclear magnetic resonance (NMR) buffer (low salt: 25-mM NaOAc-d3, pH 6.5, 25-mM NaCl, 0.1-mM ZnCl_2_ and 0.1-mM BME; high salt: same as low salt except containing 500 mM NaCl). D_2_O (10% final) was added to the dialyzed proteins before NMR experiments. Gel-shift annealing assays were performed as described below to confirm that the NC proteins obtained using different methods were all active (Supplementary Figure S1).

The peptide derived from HIV-1 CA helix 11, corresponding to residues 211–227, was purchased from GenScript (Piscataway, NJ, USA) and the p6 peptide, corresponding to residues 72–123 of NCp15 (Figure 1A), was synthesized by New England Peptide. These two peptides were purchased as HPLC-purified products and their identity was confirmed by mass spectrometry. Polyglutamic acid was purchased from Sigma-Aldrich (St. Louis, MO, USA) and used without further purification.

Unmodified human tRNA^Lys3^ (hereafter referred to as human tRNA^Lys3^) and a 105-nt RNA construct that includes the PBS of the HIV-1 genome (shortPBS) (70,71) were prepared by *in vitro* transcription (72). The RNA concentrations were determined using the following extinction coefficients: tRNA^Lys3^: 664 700 M^−1^ cm^−1^, shortPBS: 935 700 M^−1^ cm^−1^. ^32^P-labeled human tRNA^Lys3^ used in annealing assays was prepared using the same method but with α-[^32^P]-guanosine-5'-triphosphate (GTP) (Perkin Elmer, Shelton, CT, USA) instead of GTP.

Fluorescently-labeled shortPBS was prepared by labeling purified RNA at the 3′ end with fluorescein-5-thiosemicarbazide as described (73,74). A 40-nt single-stranded (ss) deoxyribonucleic acid (DNA) used in salt titration experiments was purchased from Integrated DNA Technologies (Coralville, IA, USA): 5′-FAM-ACT GGA CCA AAT TCT AAT GTG TGT GTA CGA AGC ATA GCG G-3′. A 64-nt long DNA complementary to the HIV-1 trans-activation response region (cTAR), labeled with 5′-AlexaFluor488 (AF) and 4-(4′-dimethylaminophenylazo)benzoic acid (DABCYL), was purchased from TriLink Biotechnologies (San Diego, CA, USA) (5′-AF-cTAR-DABCYL-3′) (50). A singly-labeled 5′-AF-cTAR DNA was also obtained.

### Fluorescence anisotropy binding experiments and salt titration assays

The fluorescence anisotropy (FA) binding experiments were carried out as described (75). Fluorescein-labeled shortPBS (19.2 nM) was incubated with various concentrations of NC (0–3 μM) in 50 mM HEPES, pH 7.5, 5-mM dithiothreitol (DTT), 1-mM MgCl_2_ and 20-mM NaCl. The reactions were incubated in the dark at room temperature for 30 min before measuring FA using a SpectraMax M5 plate reader (Molecular Devices, Sunnyvale, CA, USA). The data were analyzed as described, using a 1:1 binding model (75).

Salt-titration binding assays were carried out as described (76). Briefly, a fixed amount of NC (100 nM or 400 nM) was incubated with 40-nt ssDNA (20–30 nM) in the presence of increasing NaCl concentrations (0 mM to 1 M) in 20-mM HEPES, pH 7.5, 20-μM TCEP, 5-mM BME and 1-mM MgCl_2_. The reactions were incubated at room temperature in the dark for 30 min prior to FA measurements. To correct for the change of FA at high salt due to increased solution viscosity and/or DNA conformation changes, FA of control reactions in the absence of NC was also measured and the results were subtracted from the reactions with proteins. The data were analyzed as described (76). The dissociation constant, *K_d_*, varies with sodium ion concentration as follows:

(1) \begin{equation*} K_d = K_{d(1 {\rm M})} \cdot [Na]^{Z_{\rm eff} } , \end{equation*}

where *Z*_eff_ is the effective charge of NC, which corresponds to the number of charges involved in direct NA interaction. *K*_*d*(1 M)_ denotes the dissociation constant of the protein in a buffer with 1-M NaCl when all electrostatic interactions have been screened out.

### Gel-shift annealing assays

Time-course gel-shift annealing assays were performed as described (29,77). Briefly, 10-nM ^32^P-labeled human tRNA^Lys3^ was mixed with 25-nM HIV-1 shortPBS in a buffer containing 50 mM HEPES pH 7.5, 5-mM DTT, 1-mM MgCl_2_ and 20-mM NaCl. The reaction was initiated by adding NC to a final concentration of 600 nM (unless otherwise indicated). Aliquots were removed at different time points and quenched with 1% sodium dodecyl sulfate (SDS). Following two phenol–chloroform extractions to remove proteins, reactions were run on SDS-polyacrylamide gel electrophoresis to separate the annealed product from the free tRNA^Lys3^. The gels were analyzed on a Typhoon Trio Variable Mode Imager (GE Healthcare). Rate constants were calculated by fitting the data to a single-exponential equation.

To determine the effect of specific peptides (p6, CA helix 11 or polyglutamic acid) on NCp7-facilitated annealing, peptide concentration-dependent tRNA/shortPBS annealing assays were performed similar to the time-course assay. In these assays, 10-mM NaCl was used to promote NCp7–peptide interaction. The annealing reactions were performed in the presence of increasing concentrations of peptide (0–5 μM) and 800 nM of NCp7, at room temperature for 15 min prior to quenching with 1% SDS (NCp7 and peptides were mixed first to allow complex formation).

### Sedimentation assays

Sedimentation assays were used to evaluate the aggregation capability of NC proteins (29). The RNA concentrations and buffer conditions were the same as in the time-course annealing assays, except the non-ionic detergent, Tween 20 (Sigma), was added to 0.1% in some experiments. Various amounts of NC were added to the reactions, which were incubated at room temperature for 30 min to 2 h prior to centrifugation at 10 000 × *g* for 10 min. The radioactivity remaining in the supernatant was quantified by scintillation counting. The percentage of aggregated NA (*y*) versus protein concentration (*x*) was fit to the Boltzmann equation:

(2) \begin{equation*} y = \frac{{A_1 - A_2 }}{{1 + e^{\frac{{x - x_0 }}{{dx}}} }} + A_2 , \end{equation*}

Where *A*_1_ is set to 0 (the NA sedimentation before adding any proteins), *A*_2_ is the maximum percentage of NA aggregated, *dx* is the protein concentration change required for complete sedimentation and *x*_0_ is the protein concentration at which half of the NA is sequestered by NC.

### Dynamic light scattering measurements

Dynamic light scattering (DLS) measurements were carried out in 50 mM HEPES pH 7.5, 5-mM DTT, 1.3-mM MgCl_2_ and 20-mM NaCl using 20-nM tRNA^Lys3^ and 50-nM shortPBS. Measurements were performed 30 min after 1.2-μM NC addition on a Zetasizer Nano-ZS (Malvern Instruments Ltd, Worcestershire, UK) and analyzed by the Dispersion Technology Software provided.

### TAR hairpin destabilization assays

TAR hairpin destabilization experiments were performed similar to those previously described (29,50,78). Briefly, 100 nM of 5′-AF-cTAR-DABCYL-3′ was incubated with increasing concentrations of NC, in a buffer containing 50 mM HEPES, 1-mM MgCl_2_, 100-mM NaCl, 10-μM TCEP and 5-mM BME. After a 30-min incubation at room temperature, fluorescence intensity was recorded on a SpectraMax M5 plate reader and fluorescence lifetime measurements were performed on a LifeSpec (red) time-resolved spectrometer (Edinburgh Instruments, Livingston, UK). The fluorescence lifetime data were fit to a triple-exponential decay model to obtain the fluorescence lifetimes (*τ*) and populations of three states: *B*_1_, *B*_2_ and *B*_3_, corresponding to three distinct conformations (closed state: *τ*_1_ ∼ 0.2 ns, semi-open state: *τ*_2_ ∼ 1 ns and open state: *τ*_3_ ∼ 3.5 ns) as described (78). Similar analysis for 5′-AF-cTAR was carried out and fitted to a two-states model. By taking into account the fluorescence intensity data, the population of a dark state (α_0_) can be estimated as follows (78):

(3) \begin{equation*} \alpha _0 = 1 - \frac{{ \langle \tau \rangle _s }}{{ \langle \tau \rangle _{\exp } \cdot \frac{{{\rm Fl}_s }}{{{\rm Fl}_{\exp } }}}}, \end{equation*}

where <*τ>*_s_ and <*τ>*_exp_ are the mean fluorescence lifetimes for singly- and doubly-labeled species, respectively, and Fl_s_ and Fl_exp_ are the fluorescence intensities for singly- and doubly-labeled species, respectively. The value of <*τ>* can be calculated by

(4) \begin{equation*} \langle \tau \rangle = \sum {B_i \tau _i }. \end{equation*}

The dark state value (α_0_) was used to correct the values of the populations of hairpin (*B_i_*) residing in the three fluorescent states as follows:

(5) \begin{equation*} \alpha _i = B_i (1 - \alpha _0 ). \end{equation*}

### NMR data collection

^15^N/^13^C-labeled NCp7 (0.4 mM) and NCp15 (0.2 mM) in 25-mM NaOAc-d3, pH 6.5, 25-mM NaCl, 0.1-mM ZnCl_2_ and 0.1-mM BME (low salt buffer) (or in high salt buffer: same as low salt except containing 500-mM NaCl) were placed into 5-mm NMR tubes. All spectra were collected at 25°C with a Bruker Avance 800-MHz (^1^H) NMR spectrometer equipped with z-axis gradient cryogenic probeheads. Backbone resonance assignments were made with standard heteronuclear triple resonance experiments (79). All data were processed with NMRPipe (80). Resonance assignments were made with NMRView (81). The chemical shift differences were calculated based on

(6) \begin{eqnarray*} \begin{array}{*{20}c} {\Delta = } {\sqrt {\begin{array}{*{20}c} {\left( {\delta _{{\rm H}({\rm NCp7})} - \delta _{{\rm H}({\rm NCp15})} } \right)^2 + } \\ {\left( {0.15 \times \left( {\delta _{{\rm N}({\rm NCp7})} - \delta _{{\rm N}({\rm NCp15})} } \right)} \right)^2 } \\ \end{array} } } \\ \end{array} , \end{eqnarray*}

where *δ*_H(NCp7)_ and *δ*_H(NCp15)_ are the hydrogen chemical shifts and *δ*_N(NCp7)_ and *δ*_N(NCp15)_ are the nitrogen chemical shifts of NCp7 and NCp15, respectively. The value 0.15 was a scaling factor used to correct for the larger range of nitrogen chemical shift.

### Single-molecule DNA stretching studies

We used dual beam optical tweezers to stretch single bacteriophage λ DNA molecules, labeled with biotin on both 5′ ends, as previously described (82). Stretching experiments were performed in 10-mM HEPES, 50 mM Na^+^, pH 7.5. After attachment of one DNA molecule, buffer was used to rinse out the other DNA molecules, and solutions containing specific protein concentrations were flowed around the DNA to investigate protein effects on DNA stretching curves. Both the DNA stretch and release force-extension curves in the presence of protein were then recorded at pulling rates in the range of 20–500 nm/s.

### Production of mutant and wild-type HIV-1 and infectivity analysis

Mutant and wild-type (WT) proviral plasmids described above were transfected into 293T cells as described (7). Virions were analyzed for single-round infectivity on TZM-bl cells (obtained through the NIH AIDS Research and Reference Reagent Program, Division of AIDS, NIAID, NIH from Dr. John C. Kappes, Dr. Xiaoyun Wu and Tranzyme Inc.) as described previously (83).

## RESULTS

### NA binding properties

**Figure 2.**
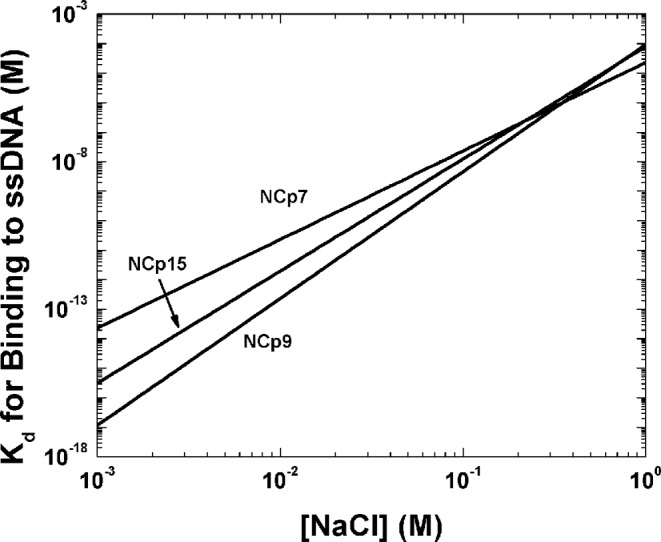
Graph showing the dependence of dissociation constants (*K_d_*) on NaCl concentration for NCp7, NCp9 and NCp15. The binding was studied using a 40-nt ssDNA.

The NA binding affinity of HIV-1 NC proteins was studied by FA. All three forms of NC bind the 105-nt shortPBS RNA sequence derived from the PBS region of the HIV-1 genome (71) with very similar apparent *K_d_* values (Table 1). To further characterize and compare the NA binding properties of NCp7, NCp9 and NCp15, salt titration binding experiments were carried out (76). In these experiments, NC proteins were first incubated with a fluorescently-labeled 40-nt ssDNA and then NaCl was used to displace NC from the oligonucleotide. ssDNA was used as a model system to avoid any complications of binding due to NA folding. The non-electrostatic binding component, *K*_*d*(1 M)_, of these three NC proteins is very similar, ∼10^−5^ M (Table 1), as expected for the proteins sharing the same ZF structures. However, as expected based on differences in overall p*I*, slight differences in the effective charge (*Z*_eff_) were observed. The *Z*_eff_ of NCp7 is ∼3.0, meaning that on average three Na^+^ cations are released from the NA phosphate upon protein binding. NCp9 possesses three more basic residues in the SP2 region (Figure 1A), but the effective charge only increases by 1.3. Interestingly, the presence of acidic residues in the p6 region (Figure 1A) reduces the effective charge of NCp15 by 0.5 relative to NCp9. Thus, in the context of NCp15 the negative charges on the p6 domain may effectively neutralize some positive charges on NCp9. The effect of *Z*_eff_ and *K*_*d*(1M)_ can be better visualized by calculating the dependence of the *K_d_* on salt (Figure 2). NCp7, NCp9 and NCp15 show very similar binding affinity to the ssDNA at 1-M NaCl. At lower salt concentrations, the differences between these NC variants become significant. NCp9 binds with the highest affinity at low salt concentration consistent with the strongest electrostatic interactions, whereas NCp7 is the weakest binder among the three (Figure 2). However, in physiological salt of ∼150 mM NaCl, the dissociation constants of all three NC proteins differ by only a few fold.

**Table 1.** Binding parameters measured for HIV-1 NCp7 and precursor proteins

	*K_d_* (nM)^a^	*K*_*d*(1 M)_ (M)^b^	*Z*_eff_^b^
NCp7	231 ± 30	(2.3 ± 0.7)·10^−5^	3.0 ± 0.3
NCp9	240 ± 18	(9.2 ± 6.1)·10^−5^	4.3 ± 0.7
NCp15	278 ± 36	(7.8 ± 4.1)·10^−5^	3.8 ± 0.7

^a^Determined from FA assays using 105-nt shortPBS in 50 mM HEPES, pH 7.5, 5-mM DTT, 1-mM MgCl_2_ and 20-mM NaCl.

^b^Determined from salt titration assays using 40-nt ssDNA. *K*_*d*(1 M)_ and *Z*_eff_ are defined as described in the text.

Values are the average of at least three measurements with the standard deviation indicated.

### NA annealing properties

**Figure 3.**
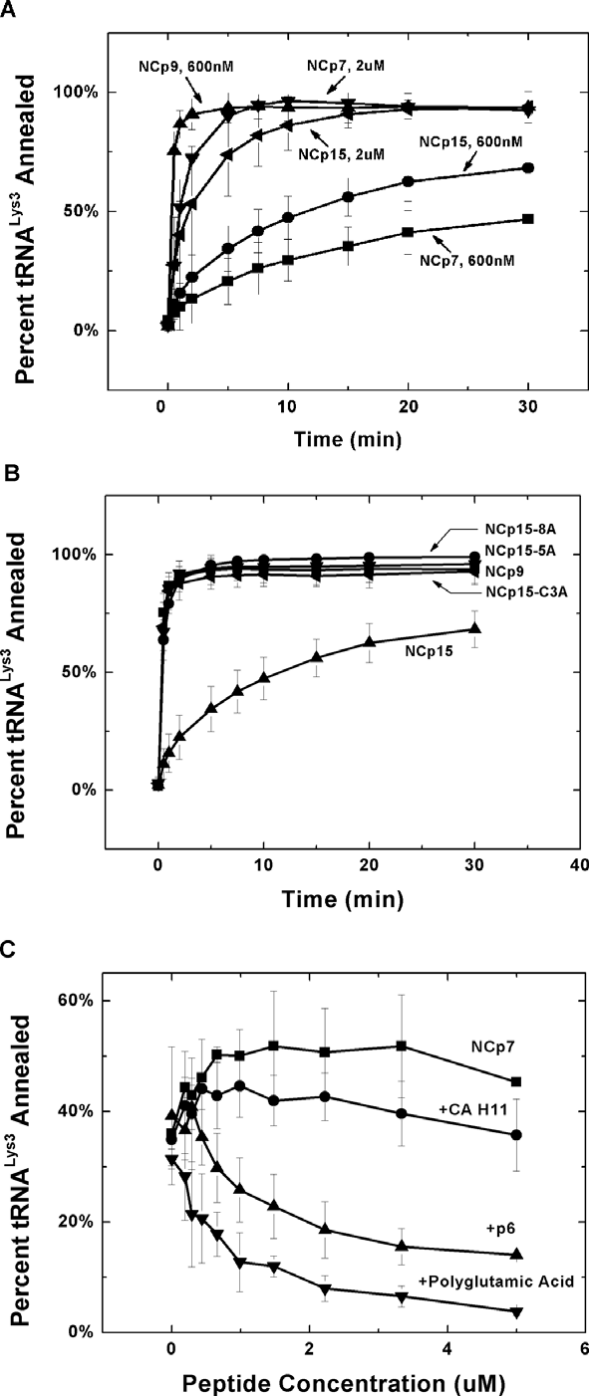
Annealing of 10-nM human tRNA^Lys3^ to 25-nM HIV-1 shortPBS as a function of time in the presence of different forms of HIV-1 NC. (**A**) Comparison of the kinetics of annealing in the presence of the indicated concentration of NCp7, NCp9 and NCp15. (**B**) Comparison of the annealing kinetics in the presence of 600-nM NCp9, WT NCp15 and NCp15 acidic residue variants. (**C**) Effect on annealing of negatively charged peptides HIV-1 p6 and polyglutamic acid or neutral peptide CA helix 11.

We next compared the NA annealing activities of NCp7, NCp9 and NCp15 (29,71,77). In these assays, ^32^P-labeled *in vitro* transcribed human tRNA^Lys3^ (76 nt) was incubated with shortPBS (105 nt) in the presence of different forms of HIV-1 NC (Figure 1). Time-course annealing assays at 600-nM protein concentration showed that NCp15 can anneal human tRNA^Lys3^ to the shortPBS at a similar rate as the mature NCp7 and the reactions reach similar extents of annealed product (Figure 3A and Table 2; gel examples are shown in Supplementary Figure S2). Surprisingly, HIV-1 NCp9 is the best annealing agent among the three forms of HIV-1 NC. However, increasing the protein concentration of NCp7 and NCp15 to 2 μM resulted in increased rates and extents of annealing that approached the level of NCp9 (Figure 3A and Table 2). Immunoblots of samples taken from WT HIV-1 failed to detect NCp15 in rapid harvest (30 min) or 46-h virus (Supplementary Figure S3). In contrast, whereas 46-h virus contained only NCp7, rapid harvest virus particles contained a mixture of NCp7 and what appears to be NCp9 as the migration distance is similar to that of purified recombinant NCp9 (Supplementary Figure S3). Based on these findings in rapid harvest virus, preliminary annealing assays with mixtures of NC protein were also carried out. Adding NCp7 or NCp15 (600 nM or 2 μM) to reactions containing 600-nM NCp9 does not negatively affect annealing (Supplementary Figure S4).

**Table 2.** Annealing rate constants of HIV-1 NC variants

NC variant, concentration	Final % annealing^a^	*k*_obs_ (min^−1^)^a^	Fold decrease^b^
NCp9, 600 nM	93.3 ± 0.5	3.05 ± 0.18	-
NCp7, 600 nM	50.4 ± 1.8	0.08 ± 0.01	38
NCp15, 600 nM	66.6 ± 3.4	0.14 ± 0.02	22
NCp15-5A, 600 nM	94.8 ± 0.3	2.38 ± 0.06	1.3
NCp15-C3A, 600 nM	91.2 ± 0.4	2.70 ± 0.16	1.1
NCp15-8A, 600 nM	98.0 ± 0.6	1.48 ± 0.14	2.1
NCp7, 2 μM	95.5 ± 0.1	0.64 ± 0.02	4.8
NCp15, 2 μM	93.0 ± 0.9	0.34 ± 0.05	9.0

^a^Final % annealing and *k*_obs_ were obtained by fitting annealing assay data to a single-exponential equation.

^b^The fold decrease was calculated relative to the rate constant for NCp9 at 600 nM.

### NA destabilization properties

**Figure 4.**
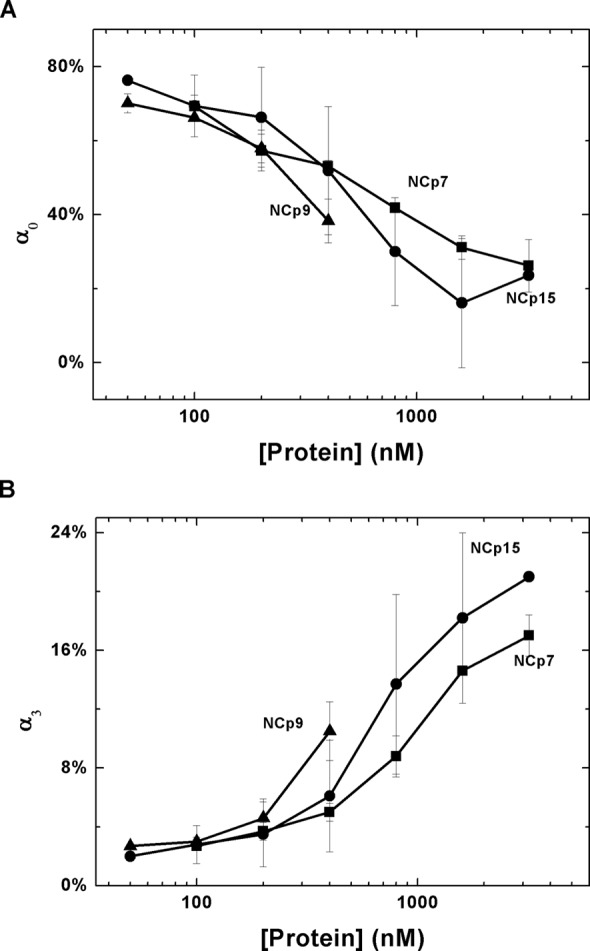
cTAR destabilization capability of HIV-1 NC. Changes in the population of the dark state (α_0_) (**A**) and the most opened state (α_3_) (**B**) of cTAR as a function of NC protein concentrations.

The NA chaperone function of retroviral NC proteins depends on two major properties—the capability of the protein to destabilize stable duplex structures and to aggregate NAs (14,16,17,24). Fast NA interaction kinetics also plays a role in chaperone function (84). To further characterize the chaperone activity of NC, Förster resonance energy transfer-based cTAR DNA hairpin destabilization assays were adopted to evaluate HIV-1 NC's duplex destabilization activity (29,50,78). When NC interacts with cTAR, up to four populations of DNA can be detected [a ‘dark state’ (α_0_) and three more open states (α_1_, α_2_, α_3_)]. The ‘dark state’ is completely closed and becomes less populated with increasing binding of NC, while the open states become more populated as a result of NC's preferential binding to ssDNA. Figure 4 shows a plot of α_0_ (Figure 4A) and α_3_, which is the most open state (Figure 4B), as a function of NC concentration. Based on these data, we conclude that the three forms of NC show comparable duplex destabilization activity. High concentrations of NCp9 induced aggregation of the DNA and thus the destabilization activity of NCp9 cannot be studied at the highest protein concentrations. (Supplementary Figure S5 summarizes the population shifts observed at the highest NC concentration used for all three forms of NC.)

### NA aggregation properties

**Figure 5.**
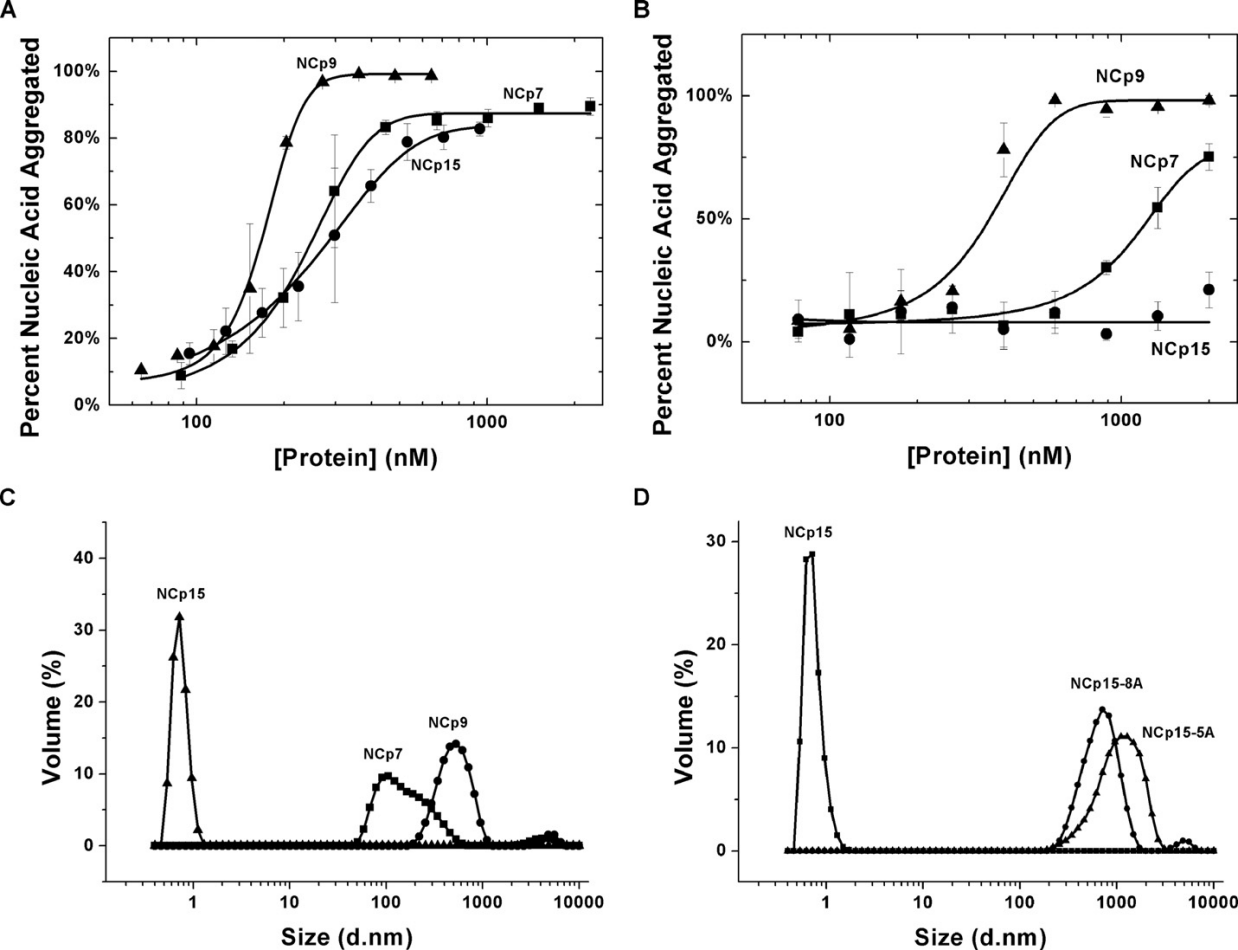
NA aggregating capabilities of different forms of HIV-1 NC. Results of sedimentation assays performed in the absence of Tween 20 (**A**) and in the presence of 0.1% Tween 20 (**B**), showing the percent of pelleted NA as a function of increasing protein concentration. (**C**) The NC–NA aggregate size distributions as determined by DLS for NCp7, NCp9 and NCp15. (**D**) Effect of neutralizing the C-terminal acidic residues of NCp15 on aggregate size distributions.

Sedimentation assays were performed to compare the NA aggregation capabilities of HIV-1 NCp7, NCp9 and NCp15. In initial studies performed in the absence of the non-ionic detergent Tween 20, the same trend was observed as in the annealing assays (Figure 5A). NCp9 is the most effective agent, requiring the least amount of protein to aggregate half of the NA used in the reaction (*C*_1/2_ ∼ 150 nM). NCp15 and NCp7 appeared to show similar aggregation activity (*C*_1/2_ ∼ 240 nM). However, in the absence of centrifugation, the presence of NCp15 depleted NA in solution (data not shown), suggesting that the apparent sedimentation observed is due to adsorption of NCp15–NA complexes to the sides of the microcentrifuge tubes used. When experiments were performed in the presence of 0.1% Tween 20, the effect was eliminated and NCp15's aggregation capability was measured as negligible (Figure 5B). Since the sedimentation assay does not assess aggregate morphology, DLS was used to study the aggregate size distribution of all three forms of NC. Interestingly, whereas NCp7 and NCp9 can form stable, large protein–RNA aggregates that scatter light effectively, the NCp15–RNA complexes scatter light very weakly, resulting in small apparent sizes (Figure 5C). In fact, RNA alone or NCp15 alone show similar calculated size distributions as NCp15–RNA complexes (Supplementary Figure S6). Although the apparent aggregate size may not reflect the actual dimensions of NCp15–NA complexes, these data strongly support the formation of very different complex morphology in the case of NCp15. The calculated aggregate size distribution indicates that the sizes of complexes formed by all forms of NC vary, with NCp9>NCp7>>NCp15.

### Role of the p6 domain in regulating the chaperone function of NCp15

We hypothesize that the acidic nature of the p6 domain may play a role in distinct NA interaction properties of NCp15. To test this hypothesis, several mutant forms of NCp15 were generated containing three (C3A), five (5A) or eight (8A) E/D to A mutations (Figure 1A). DLS analysis revealed that neutralizing five or eight acidic residues resulted in protein–NA aggregates that were similar in size distribution to those of NCp9 (Figure 5D).

As shown in Figure 3B, neutralizing any number of acidic residues in p6 also restored annealing activity to the level of NCp9 (Table 2). Consistent with the negative impact of the acidic residues on annealing activity, a synthetic p6 peptide added to NCp7 *in trans* significantly inhibited NC's annealing activity of NCp7 (Figure 3C). A negatively charged polyglutamic acid inhibited NCp7 annealing activity even more strongly than p6, consistent with the higher negative charge density on the polyglutamic acid, while a neutral peptide derived from HIV-1 CA's CTD (85) does not have a significant effect (Figure 3C). Taken together, these data show that the acidic residues in p6 modulate both the aggregation and annealing properties of NCp15.

**Figure 6.**
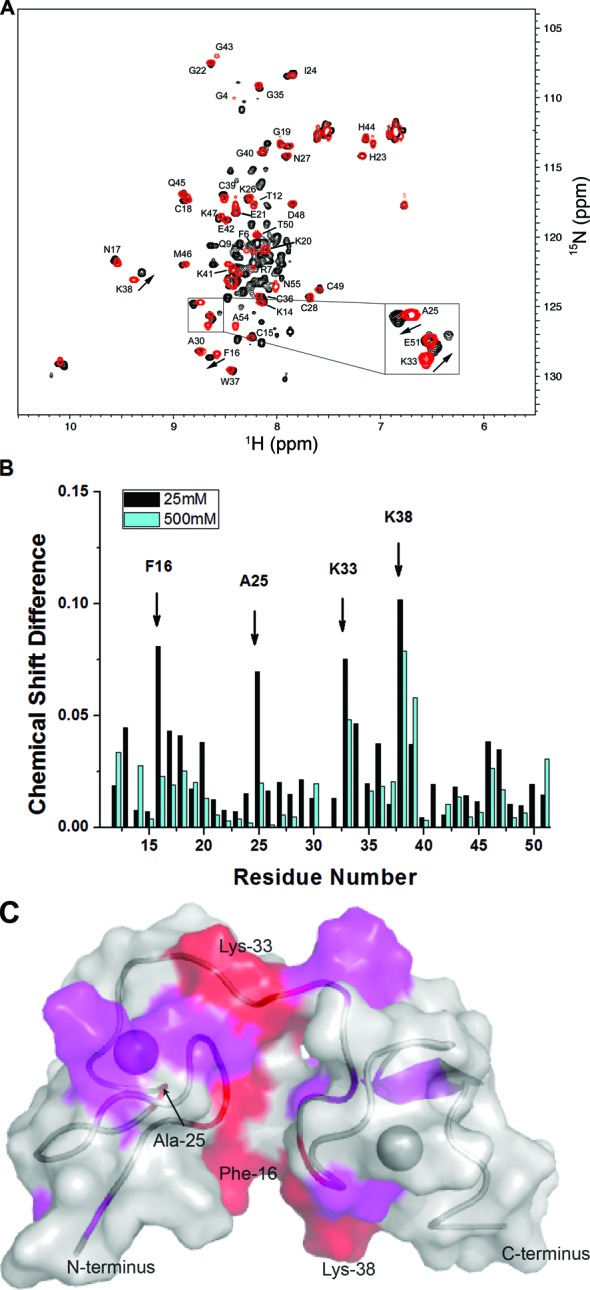
^15^N-^1^H HSQC NMR analysis of NCp7 and NCp15. (**A**) HSQC spectra collected in low salt (25-mM NaCl) NMR buffer for NCp7 (red) and NCp15 (black). (**B**) Plot of the chemical shift differences between NCp7 and NCp15 versus residue number for the ZF region at 25-mM NaCl (black) and 500-mM NaCl (dark cyan). The most significantly perturbed residues are labeled. (**C**) Chemical shift differences between NCp7 and NCp15 observed at 25-mM NaCl shown on the NCp7 structure (pdb: 1ESK). Red regions indicate chemical shift changes greater than 0.05 ppm. Magenta regions indicate changes greater than 0.025 ppm but smaller than 0.05 ppm. Zinc ions are shown as spheres. The backbone of NC is shown as ribbons.

The NMR structure of the p6 domain alone shows that it is a largely unstructured peptide with two short helical segments forming in the presence of trifluoroethanol or in dodecylphosphocholine micelle solution (52,86). To determine whether p6 covalently appended to NCp9 results in a fold-back conformation as previously observed for HTLV-1 NC (50,51), ^15^N-/^13^C-labeled NCp7 and NCp15 were prepared and backbone chemical shifts were determined with conventional triple resonance experiments. Figure 6A shows an overlay of the Heteronuclear Single Quantum Coherence (HSQC) spectra of NCp7 (red) and NCp15 (black) recorded in 25-mM NaCl. While most signals corresponding to the NCp7 domain in the context of NCp15 generally overlay very well with their counterparts in free-standing NCp7, some peaks are clearly perturbed (Figure 6A). Four residues (F16, A25, K33 and K38) in the ZFs show chemical shift differences greater than 0.05 ppm when comparing NCp7 and NCp15 (Figure 6B). These residues are close in space in the three-dimensional structure (69,87) (Figure 6C). In the presence of 500-mM NaCl, all of these chemical shift differences were significantly reduced in magnitude (Figure 6B). Consistent with our NMR results, at higher salt (100-mM NaCl), the annealing rate differences observed between NCp9 and NCp15 are reduced, and at 250-mM NaCl, the annealing rates of NCp9 and NCp15, although both low, are identical (Supplementary Figure S7).

### Single-molecule DNA stretching studies

**Figure 7.**
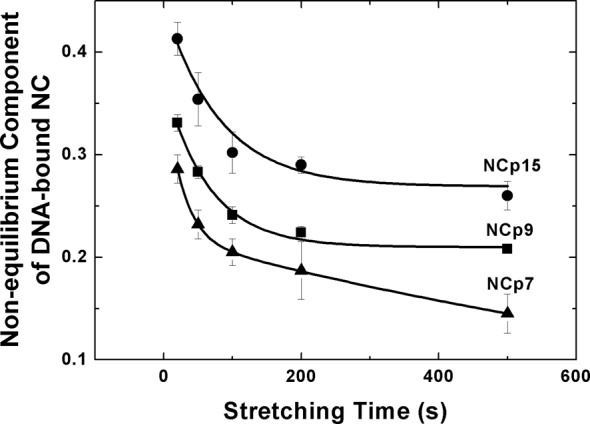
Non-equilibrium component of DNA-bound HIV-1 NC molecules as a function of the DNA stretching time at 20-nM protein concentration. The non-equilibrium component was obtained from the hysteresis of the NC-DNA stretch-release cycle as a function of pulling rate normalized by the area between the force-extension curves for the protein-free dsDNA and protein-saturated DNA (89). The lines are fits to Equation (7) with the fit parameters summarized in Supplementary Table S1.

We next used single-molecule DNA stretching (50,75,88,89) to probe the differences in NA interaction kinetics of HIV-1 NCp7, NCp9 and NCp15. Typical DNA stretch-release cycles in the presence of 20 nM of each of the HIV-1 NC proteins performed at two different pulling rates (50 and 500 nm/s) are presented in Supplementary Figure S8. All three proteins lengthen the DNA at high forces due to intercalation (90–92), while also destabilizing the DNA duplex (88). As with all intercalators, increasing the force promotes additional binding and decreasing the force promotes protein dissociation. Therefore, the amount of NC bound is less than the equilibrium amount during stretching and more than the equilibrium amount during release, resulting in disagreement between the stretch and release curves, or hysteresis. Because the maximum hysteresis would be obtained for an infinitely slow protein that does not have time to bind during DNA stretching or to dissociate during release, we normalize our measured hysteresis by this maximum value to obtain the non-equilibrium component of NC binding. The hysteresis is quantified in Figure 7, which shows the non-equilibrium component of the DNA-bound NC molecules as a function of DNA stretching time, or the time it takes for a single stretch and release cycle at a given pulling rate. This non-equilibrium fraction represents the fraction of NC bound to DNA that exhibits binding kinetics slower than the time scale of stretching. For all measured stretching times, the non-equilibrium component is at least 44% greater for NCp15 relative to NCp7.

The time behavior of this non-equilibrium DNA-bound NC component, *F*(*t*), exhibits multiple time scales, most likely due to the continuum of the sequence-dependent NC-DNA binding sites. *F*(*t*) can be minimally fit to a double exponential function

(7) \begin{equation*} F(t) = f_{\rm fast} \cdot e^{ - t/\tau _{\rm fast} } + f_{\rm slow} \cdot e^{ - t/\tau _{\rm slow} } , \end{equation*}

where *f*_fast_, *f*_slow_ and *τ*_fast_, *τ*_slow_ are the fast and the slow fractions of the non-equilibrium NC binding component and the corresponding relaxation times, respectively. The resulting parameters are given in Supplementary Table S1. The non-equilibrium components of the DNA-bound NC molecules have fast fractions varying from 0.14 to 0.19, with relaxation times ranging from 25 to 47 s, and slow fractions with relaxation times in the range of 1000–5000 s. In addition to having a larger non-equilibrium component for all stretching times, NCp15 has a significantly larger slow fraction of ∼0.3, relative to ∼0.2 for NCp7 and NCp9.

### Cell-based assays

Cell-based assays were carried out to determine the effect of neutralizing the acidic residues of p6 on viral infectivity. In the context of otherwise WT virus, the 8 D/E to A changes (see Figure 1) reduced viral infectivity by ∼20-fold in single-round TZB-bl infectivity assays [(83), Table 3], most likely because mutations disrupt other essential function of p6. Moreover, an HIV-1 mutant containing NCp15-8A failed to rescue viral infectivity of the NCp15-containing virus, which displayed only 1% infectivity compared to WT HIV-1; the infectivity of NCp15-8A was ∼3-fold lower than that of NCp15_WT_. However, it is apparent that these alterations did affect other properties of the mutant viruses. For example, in the case of the NCp15-8A virus, an additional protease cleavage site was introduced upon mutating the 8 D/E to A residues (Supplementary Figure S9); thus, the reduction in infectivity may be due to these other factors and not necessarily elimination of ionic interactions between the p6 domain and NC. This reinforces the importance of *in vitro* studies using recombinant proteins, since hypotheses are occasionally not directly demonstrable in the virus/cell setting due to unforeseen effects of mutations on viral processes.

**Table 3.** Single-round infectivity analysis of WT and mutant HIV-1

Virus	Relative infectivity (% of WT)^a^	Relative infectivity (% of NCp15_WT_)^b^
(-)-Control^c^	0	0
WT	100	NA
NCp15_WT_	0.96 ± 0.25	100
NCp15_(PTAP to LIRL)_	0.073 ± 0.052	6.4 ± 4.3
NCp15_(FRFG to SRSG)_	0.65 ± 0.41	79 ± 9
NCp15_(PTAP to LIRL/FRFG to SRSG)_	0.11 ± 0.05	10 ± 4
WT-8A^d^	6.2 ± 4.7	NA
NCp15-8A^d^	0.20 ± 0.01	29 ± 1

^a^Single-round TZB-bl infectivity assays were performed as described previously (83). Averages ± standard deviations of exogenous template reverse transcriptase (RT) activity-normalized titers are reported as % of WT HIV-1 from two to four independent infection experiments.

^b^Averages ± standard deviations of exogenous template RT activity-normalized titers are reported as % of NCp15_WT_ HIV-1 from two to four independent infection experiments. NA is not applicable.

^c^Infectivity of supernatants from mock-transfected 293T cells.

^d^The 8A variants contain eight acidic residue changes to alanine in p6, as shown in Figure 1.

*In vivo*, the p6 domain has been reported to interact with many other cellular and viral proteins (52). These interactions may be detrimental to HIV-1 in the context of uncleaved NCp15. To test this hypothesis, two sets of mutations were designed in the context of NCp15-containing virus to eliminate potential interactions with Tsg101 and Vpr. Mutation of the PTAP motif at residues 78–81 of NCp15 to LIRL was designed to disrupt the interaction between p6 and Tsg101 (53,93), while changing the FRFG motif at residues 86–89 of NCp15 to SRSG should eliminate p6–Vpr interaction (66). An HIV-1 clone with both motifs mutated was also generated. Single-round infectivity assays were carried out using a TZM-bl cell line (83). If the interactions between NCp15 and Tsg101 or Vpr have negative effects on viral replication, eliminating these motifs should rescue the viral infectivity defect observed in an HIV-1 variant expressing NCp15. However, all of these mutations fail to show significant increases in infectivity compared to the virus expressing NCp15 without the secondary changes (Table 3). Thus, these p6 interactions do not appear to be the major cause of the large decreases in viral infectivity observed with NCp15 containing HIV-1.

## DISCUSSION

Previous cell-based assays showed that HIV-1 processing mutants that contain NCp9 resulted in variable effects on viral infectivity (43–48). These variations are likely due to the different mutations and HIV-1 strains used. In contrast, processing mutations that result in NCp15-containing virus consistently led to large defects in viral infectivity and replication (43,45,46). Lending credence to the transient presence of some of these precursors in WT virus, we observed an α-NCp7-reactive band in an immunoblot of rapid harvest virus that migrates similarly to purified recombinant NCp9, along with NCp7 (Supplementary Figure S3), but disappears on longer incubation of virus. On the other hand, an NCp15 species was not detectable in rapid harvest or 46-h harvest HIV-1, in agreement with previous results reported by Kaplan *et al.* (94).

In this work, to understand the strict requirement for NCp15 processing for HIV-1 replication, we compared the NA binding and chaperone functions of the three major forms of NC, mature NCp7, NCp9 and NCp15. We observed only minor differences in these *in vitro* activities, consistent with the conclusion that an NCp15-expressing virus was able to carry out reverse transcription (43,47). Considering that the effective concentration of NC is quite high in virions (∼mM), it is likely that NCp15 can carry out RNA annealing efficiently during HIV-1 infection. NCp9 actually displayed the highest activity in all the assays conducted here (NA binding, annealing, aggregation and destabilization). In a parallel study on the effects of the three NC proteins on HIV-1 reverse transcriptase reactions, Wu *et al.* also find that under optimal conditions, NCp9 has the strongest chaperone activity (T. Wu *et al.*, submitted for publication). Using a different assay system, it has been shown that although NCp9 is slower than NCp7 and NCp15 in stimulating immature genomic RNA dimerization, it is capable of facilitating mature dimer formation, while NCp15 cannot (95). While the relative levels of chaperone activity may differ, depending on the assay used, the *in vitro* activity differences cannot fully explain the dramatic infectivity decreases observed for HIV-1 NCp15-containing virus.

Sedimentation assays and DLS experiments showed that NCp7 and NCp9 both promote the formation of large protein–RNA aggregates. In contrast, NCp15–RNA complexes could not be pelleted (in the presence of 0.1% Tween 20) and failed to scatter light effectively by DLS (Figure 5C). This is in accord with a previous electron microscopy study showing that NCp15 binds ssDNA like ‘beads on a string’, unlike NCp7 or NCp9, which form condensed aggregates (96,97).

The distinct NA interaction mode of NCp15 is regulated by the acidic p6 domain. The results of NMR studies performed here provide support for a fold-back conformation in solution for NCp15, wherein the acidic p6 domain interacts with the basic ZF domain. This interaction seems to be electrostatic, since at 500-mM NaCl the chemical shift perturbations are weaker. Neutralizing the C-terminal acidic residues in p6 improves NCp15's chaperone function (Figure 3B), weakens the fold-back conformation and results in larger aggregates upon NA binding (Figure 5D). Adding p6 peptide *in trans* also partially inhibits the annealing activity of NCp7 (Figure 3C).

In the NA-bound state, similar inter-protein interactions between the p6 and ZF domains of neighboring NCp15 molecules would lead to slower protein dissociation from NA. Indeed, single-molecule DNA stretching studies strongly support this idea. According to the observed DNA stretching hysteresis (Supplementary Figure S8), most of the DNA-bound NC molecules are in equilibrium on the time scales of 10–100 s, as previously observed (84). These DNA-bound NC molecules behave like mobile multivalent cations, optimizing their positions to maximize electrostatic self-attraction (98,99) and aggregate density (96,100–103). Compared to NCp7 and NCp9, NCp15 has a larger overall non-equilibrium fraction of DNA-bound NC molecules. It also has a larger fraction of DNA-bound NC molecules that dissociate on slow time scales of ∼1000 s (Figure 7 and Supplementary Table S1) and do not contribute to self-attraction and aggregation. Although the differences between the slow fractions of DNA-bound NCp7, NCp9 and NCp15 are moderate, a critical concentration of highly charged mobile cations is known to be required for NA aggregation (98). This condition appears to be satisfied for NCp7 and NCp9, but not for NCp15. The fold-back conformation observed when free in solution and the more open conformation when bound to NAs may also explain the requirement of NA binding for efficient HIV-1 protease cleavage of NCp15 at SP2/p6 (97,104). A variant of NCp15 that is defective for binding RNA cannot be processed by HIV-1 protease efficiently (105).

Similar CTD/NTD interactions have been proposed for HTLV-1 NC (50,51). HTLV-1 NC is a poor NA chaperone, but deletion of its acidic CTD restores chaperone function (50,75). The presence of the CTD also greatly slows down the NA interaction kinetics of HTLV-1 NC (50,75). Thus, for both HTLV-1 NC and HIV-1 NCp15, the acidic CTD negatively regulates chaperone function and NA binding kinetics, although the effect in the case of NCp15 is milder (50). Comparing the sequences of p6 in HIV-1 NC with the CTD of HTLV-1 NC (50,75) provides a plausible explanation for the much weaker effects of the former. It is well known that the interaction between macromolecules correlates with their charge density, but not their net charge (106,107). Indeed, the region of highest negative charge density within the CTD of HTLV-1 NC (eight residues, 57–64) contains five anionic residues. In contrast, the highest negative charge density region of p6 (15 residues, 77–91) has five negatively charged amino acids and one positive residue.

Both NCp15 and HTLV-1 NC bear some resemblance to the prototypical ssDNA binding protein of the bacteriophage T4 gp32. gp32 has a highly negatively-charged CTD, which in the unbound state binds via intramolecular interactions to the protein's cationic ssDNA binding site, thereby slowing the NA binding on rate (108–110). Upon binding to ssDNA, gp32 forms a filament and binds in an intermolecular fashion to neighboring gp32 molecules. The gp32–gp32 interactions are hydrophobic in nature and are not associated with the electrostatic CTD–ssDNA binding site interactions (111–113). Instead, in its ssDNA-bound form, the CTD of gp32 is solvent-exposed for additional interactions with regulatory proteins (114).

Prior to viral assembly, NCp15 is appended to Gag's C-terminus. If the NA interaction properties of NCp15 in the context of Gag resemble those of the processed protein, the beads-on-a-string-type interaction with the HIV-1 genome may initially prevent NA aggregation and expose the cleavage sites for processing (97,104). During maturation, the first cleavage happens between the MA-CA-SP1 domain and the NCp7-SP2-p6 domain, thereby liberating NCp15 (39). It is possible that this form of NC is unable to effectively aggregrate the RNA, whereas the next cleavage separates the p6 domain from NCp15 leading to densely aggregated gRNA–NC complexes that may be more readily packaged into the viral capsid. Based on the observations reported here, as well as previous work (96,97), the gRNA complex with unprocessed NCp15 protein is expected to have a much larger volume, which would create a significantly higher osmotic pressure when packaged into mature HIV-1 capsid. In accord with this expectation, virions with NCp15 processing defects show deformed capsid structures with significantly larger volumes than WT virions (46). This defect in mature capsid core formation appears to occur not only when NCp15 cleavage is completely blocked but also when the timing of the cleavage is altered (46), leading to incomplete NC processing prior to mature capsid assembly (40). Indeed, variations in Gag processing rates in response to protease inhibitors lead to core structure and stability changes and major infectivity defects (115–117), which further highlights the requirement for NCp15 processing prior to mature capsid formation (37,41).

Neither disrupting p6–Tsg101 nor p6–Vpr interaction could rescue viral infectivity of a SP2/p6 cleavage mutant (Table 3), showing that these interactions are likely not the cause of non-infectious virus. The results of recent studies highlight the importance of a mature core of optimal stability on viral infectivity and suggest the presence of an intact capsid structure through at least a portion of the reverse transcription process [(118) and references therein]. Moreover, variations in WT core stability have dramatic effects on reverse transcription (119), whereas inhibiting reverse transcription stabilizes the core (118,120). We propose that the NTD–CTD interaction present in NCp15 disrupts this protein's aggregation capabilities, thus leading to abnormally large volumes of NC–gRNA complexes, associated mature cores of lower stability and the observed detrimental effects on viral infectivity.

## ACCESSION NUMBER

GenBank accession number: AF324493.

## SUPPLEMENTARY DATA

Supplementary Data are available at NAR Online, including [121–123].

SUPPLEMENTARY DATA

## References

[1] Henderson L., Bowers M., Sowder R., Serabyn S., Johnson D., Bess J., Arthur L., Bryant D., Fenselau C. (1992). Gag proteins of the highly replicative MN strain of human immunodeficiency virus type 1: posttranslational modifications, proteolytic processings, and complete amino acid sequences. J. Virol..

[2] Zhang Y., Qian H., Love Z., Barklis E. (1998). Analysis of the assembly function of the human immunodeficiency virus type 1 gag protein nucleocapsid domain. J. Virol..

[3] Huseby D., Barklis R., Alfadhli A., Barklis E. (2005). Assembly of human immunodeficiency virus precursor gag proteins. J. Biol. Chem..

[4] Muriaux D., Darlix J.-L. (2010). Properties and functions of the nucleocapsid protein in virus assembly. RNA Biol..

[5] O'Carroll I., Crist R., Mirro J., Harvin D., Soheilian F., Kamata A., Nagashima K., Rein A. (2012). Functional redundancy in HIV-1 viral particle assembly. J. Virol..

[6] Aldovini A., Young R. (1990). Mutations of RNA and protein sequences involved in human immunodeficiency virus type 1 packaging result in production of noninfectious virus. J. Virol..

[7] Gorelick R.J., Nigida S., Bess J., Arthur L., Henderson L., Rein A. (1990). Noninfectious human immunodeficiency virus type 1 mutants deficient in genomic RNA. J. Virol..

[8] De Guzman R., Wu Z., Stalling C., Pappalardo L., Borer P., Summers M. (1998). Structure of the HIV-1 nucleocapsid protein bound to the SL3 psi-RNA recognition element. Science.

[9] Amarasinghe G., De Guzman R., Turner R., Chancellor K., Wu Z., Summers M. (2000). NMR structure of the HIV-1 nucleocapsid protein bound to stem-loop SL2 of the psi-RNA packaging signal. Implications for genome recognition. J. Mol. Biol..

[10] Darlix J.-L., Gabus C., Nugeyre M., Clavel F., Barré-Sinoussi F. (1990). Cis elements and trans-acting factors involved in the RNA dimerization of the human immunodeficiency virus HIV-1. J. Mol. Biol..

[11] Feng Y., Copeland T., Henderson L., Gorelick R.J., Bosche W., Levin J.G., Rein A. (1996). HIV-1 nucleocapsid protein induces “maturation” of dimeric retroviral RNA in vitro. Proc. Natl. Acad. Sci. U.S.A..

[12] Laughrea M., Shen N., Jetté L., Darlix J.-L., Kleiman L., Wainberg M. (2001). Role of distal zinc finger of nucleocapsid protein in genomic RNA dimerization of human immunodeficiency virus type 1; no role for the palindrome crowning the R-U5 hairpin. Virology.

[13] Kafaie J., Song R., Abrahamyan L., Mouland A., Laughrea M. (2008). Mapping of nucleocapsid residues important for HIV-1 genomic RNA dimerization and packaging. Virology.

[14] Rein A., Henderson L., Levin J.G. (1998). Nucleic-acid-chaperone activity of retroviral nucleocapsid proteins: significance for viral replication. Trends Biochem. Sci..

[15] Cristofari G., Darlix J.-L. (2002). The ubiquitous nature of RNA chaperone proteins. Prog. Nucleic Acid Res. Mol. Biol..

[16] Levin J.G., Guo J., Rouzina I., Musier-Forsyth K. (2005). Nucleic acid chaperone activity of HIV-1 nucleocapsid protein: critical role in reverse transcription and molecular mechanism. Prog. Nucleic Acid Res. Mol. Biol..

[17] Levin J.G., Mitra M., Mascarenhas A., Musier-Forsyth K. (2010). Role of HIV-1 nucleocapsid protein in HIV-1 reverse transcription. RNA Biol..

[18] Carteau S., Batson S., Poljak L., Mouscadet J., de Rocquigny H., Darlix J.-L., Roques B., Käs E., Auclair C. (1997). Human immunodeficiency virus type 1 nucleocapsid protein specifically stimulates Mg^2+^-dependent DNA integration in vitro. J. Virol..

[19] Carteau S., Gorelick R.J., Bushman F. (1999). Coupled integration of human immunodeficiency virus type 1 cDNA ends by purified integrase in vitro: stimulation by the viral nucleocapsid protein. J. Virol..

[20] Buckman J., Bosche W., Gorelick R.J. (2003). Human immunodeficiency virus type 1 nucleocapsid zn(2+) fingers are required for efficient reverse transcription, initial integration processes, and protection of newly synthesized viral DNA. J. Virol..

[21] Gao K., Gorelick R.J., Johnson D., Bushman F. (2003). Cofactors for human immunodeficiency virus type 1 cDNA integration in vitro. J. Virol..

[22] Poljak L., Batson S.M., Ficheux D., Roques B.P., Darlix J.-L., Käs E. (2003). Analysis of NCp7-dependent activation of HIV-1 cDNA integration and its conservation among retroviral nucleocapsid proteins. J. Mol. Biol..

[23] Thomas J., Gagliardi T., Alvord W., Lubomirski M., Bosche W., Gorelick R.J. (2006). Human immunodeficiency virus type 1 nucleocapsid zinc-finger mutations cause defects in reverse transcription and integration. Virology.

[24] Darlix J.-L., Lapadat-Tapolsky M., de Rocquigny H., Roques B. (1995). First glimpses at structure-function relationships of the nucleocapsid protein of retroviruses. J. Mol. Biol..

[25] Rajkowitsch L., Chen D., Stampfl S., Semrad K., Waldsich C., Mayer O., Jantsch M., Konrat R., Bläsi U., Schroeder R. (2007). RNA chaperones, RNA annealers and RNA helicases. RNA Biol..

[26] Watts J., Dang K., Gorelick R.J., Leonard C., Bess J., Swanstrom R., Burch C., Weeks K. (2009). Architecture and secondary structure of an entire HIV-1 RNA genome. Nature.

[27] Cen S., Huang Y., Khorchid A., Darlix J.-L., Wainberg M., Kleiman L. (1999). The role of Pr55^gag^ in the annealing of tRNA3Lys to human immunodeficiency virus type 1 genomic RNA. J. Virol..

[28] Feng Y., Campbell S., Harvin D., Ehresmann B., Ehresmann C., Rein A. (1999). The human immunodeficiency virus type 1 Gag polyprotein has nucleic acid chaperone activity: possible role in dimerization of genomic RNA and placement of tRNA on the primer binding site. J. Virol..

[29] Jones C., Datta S., Rein A., Rouzina I., Musier-Forsyth K. (2011). Matrix domain modulates HIV-1 Gag's nucleic acid chaperone activity via inositol phosphate binding. J. Virol..

[30] Basu V., Song M., Gao L., Rigby S., Hanson M., Bambara R. (2008). Strand transfer events during HIV-1 reverse transcription. Virus Res..

[31] Dorfman T., Luban J., Goff S., Haseltine W., Göttlinger H. (1993). Mapping of functionally important residues of a cysteine-histidine box in the human immunodeficiency virus type 1 nucleocapsid protein. J. Virol..

[32] Ottmann M., Gabus C., Darlix J.-L. (1995). The central globular domain of the nucleocapsid protein of human immunodeficiency virus type 1 is critical for virion structure and infectivity. J. Virol..

[33] Darlix J.-L., Cristofari G., Rau M., Péchoux C., Berthoux L., Roques B. (2000). Nucleocapsid protein of human immunodeficiency virus as a model protein with chaperoning functions and as a target for antiviral drugs. Adv. Pharmacol..

[34] Musah R. (2004). The HIV-1 nucleocapsid zinc finger protein as a target of antiretroviral therapy. Curr. Top. Med. Chem..

[35] de Rocquigny H., Shvadchak V., Avilov S., Dong C., Dietrich U., Darlix J.-L., Mély Y. (2008). Targeting the viral nucleocapsid protein in anti-HIV-1 therapy. Mini Rev. Med. Chem..

[36] Mori M., Manetti F., Botta M. (2011). Targeting protein-protein and protein-nucleic acid interactions for anti-HIV therapy. Curr. Pharm. Des..

[37] Lee S.-K., Potempa M., Swanstrom R. (2012). The choreography of HIV-1 proteolytic processing and virion assembly. J. Biol. Chem..

[38] Erickson-Viitanen S., Manfredi J., Viitanen P., Tribe D., Tritch R., Hutchison C., Loeb D., Swanstrom R. (1989). Cleavage of HIV-1 gag polyprotein synthesized in vitro: sequential cleavage by the viral protease. AIDS Res. Hum. Retroviruses.

[39] Pettit S., Moody M., Wehbie R., Kaplan A., Nantermet P., Klein C., Swanstrom R. (1994). The p2 domain of human immunodeficiency virus type 1 Gag regulates sequential proteolytic processing and is required to produce fully infectious virions. J. Virol..

[40] Wiegers K., Rutter G., Kottler H., Tessmer U., Hohenberg H., Kräusslich H. (1998). Sequential steps in human immunodeficiency virus particle maturation revealed by alterations of individual Gag polyprotein cleavage sites. J. Virol..

[41] Mirambeau G., Lyonnais S., Gorelick R.J. (2010). Features, processing states, and heterologous protein interactions in the modulation of the retroviral nucleocapsid protein function. RNA Biol..

[42] Tözsér J., Shulenin S., Louis J., Copeland T., Oroszlan S. (2004). In vitro processing of HIV-1 nucleocapsid protein by the viral proteinase: effects of amino acid substitutions at the scissile bond in the proximal zinc finger sequence. Biochemistry.

[43] Coren L., Thomas J., Chertova E., Sowder R., Gagliardi T., Gorelick R.J., Ott D. (2007). Mutational analysis of the C-terminal gag cleavage sites in human immunodeficiency virus type 1. J. Virol..

[44] Kafaie J., Dolatshahi M., Ajamian L., Song R., Mouland A., Rouiller I., Laughrea M. (2009). Role of capsid sequence and immature nucleocapsid proteins p9 and p15 in human immunodeficiency virus type 1 genomic RNA dimerization. Virology.

[45] Lee S.-K., Harris J., Swanstrom R. (2009). A strongly transdominant mutation in the human immunodeficiency virus type 1 gag gene defines an Achilles heel in the virus life cycle. J. Virol..

[46] de Marco A., Heuser A.-M., Glass B., Kräusslich H.-G., Müller B., Briggs J. (2012). Role of the SP2 domain and its proteolytic cleavage in HIV-1 structural maturation and infectivity. J. Virol..

[47] Ohishi M., Nakano T., Sakuragi S., Shioda T., Sano K., Sakuragi J.-i. (2011). The relationship between HIV-1 genome RNA dimerization, virion maturation and infectivity. Nucleic Acids Res..

[48] Dam E., Quercia R., Glass B., Descamps D., Launay O., Duval X., Kräusslich H.-G., Hance A.J., Clavel F., Group A.S. (2009). Gag mutations strongly contribute to HIV-1 resistance to protease inhibitors in highly drug-experienced patients besides compensating for fitness loss. PLoS Pathog..

[49] Cimarelli A., Luban J. (2001). Context-dependent phenotype of a human immunodeficiency virus type 1 nucleocapsid mutation. J. Virol..

[50] Qualley D., Stewart-Maynard K., Wang F., Mitra M., Gorelick R.J., Rouzina I., Williams M.C., Musier-Forsyth K. (2010). C-terminal domain modulates the nucleic acid chaperone activity of human T-cell leukemia virus type 1 nucleocapsid protein via an electrostatic mechanism. J. Biol. Chem..

[51] Morcock D., Kane B., Casas-Finet J. (2000). Fluorescence and nucleic acid binding properties of the human T-cell leukemia virus-type 1 nucleocapsid protein. Biochim. Biophys. Acta.

[52] Fossen T., Wray V., Bruns K., Rachmat J., Henklein P., Tessmer U., Maczurek A., Klinger P., Schubert U. (2005). Solution structure of the human immunodeficiency virus type 1 p6 protein. J. Biol. Chem..

[53] Huang M., Orenstein J., Martin M., Freed E. (1995). p6^Gag^ is required for particle production from full-length human immunodeficiency virus type 1 molecular clones expressing protease. J. Virol..

[54] Garrus J., von Schwedler U., Pornillos O., Morham S., Zavitz K., Wang H., Wettstein D., Stray K., Côté M., Rich R. (2001). Tsg101 and the vacuolar protein sorting pathway are essential for HIV-1 budding. Cell.

[55] Strack B., Calistri A., Craig S., Popova E., Göttlinger H. (2003). AIP1/ALIX is a binding partner for HIV-1 p6 and EIAV p9 functioning in virus budding. Cell.

[56] Fujii K., Hurley J., Freed E. (2007). Beyond Tsg101: the role of Alix in ‘ESCRTing’ HIV-1. Nat. Rev. Microbiol..

[57] Paxton W., Connor R., Landau N. (1993). Incorporation of Vpr into human immunodeficiency virus type 1 virions: requirement for the p6 region of gag and mutational analysis. J. Virol..

[58] Kondo E., Mammano F., Cohen E., Göttlinger H. (1995). The p6^gag^ domain of human immunodeficiency virus type 1 is sufficient for the incorporation of Vpr into heterologous viral particles. J. Virol..

[59] Lu Y., Bennett R., Wills J., Gorelick R.J., Ratner L. (1995). A leucine triplet repeat sequence (LXX)4 in p6^gag^ is important for Vpr incorporation into human immunodeficiency virus type 1 particles. J. Virol..

[60] Romani B., Engelbrecht S. (2009). Human immunodeficiency virus type 1 Vpr: functions and molecular interactions. J. Gen. Virol..

[61] Fritz J.V., Briant L., Mély Y., Bouaziz S., de Rocquigny H. (2010). HIV-1 viral protein r: from structure to function. Future Virol..

[62] Zhao R., Li G., Bukrinsky M. (2011). Vpr-host interactions during HIV-1 viral life cycle. J. Neuroimmune Pharmacol..

[63] Accola M.A., Ohagen A., Gottlinger H.G. (2000). Isolation of human immunodeficiency virus type 1 cores: retention of Vpr in the absence of p6gag. J. Virol..

[64] Welker R., Hohenberg H., Tessmer U., Huckhagel C., Kräusslich H. (2000). Biochemical and structural analysis of isolated mature cores of human immunodeficiency virus type 1. J. Virol..

[65] Adachi A., Gendelman H., Koenig S., Folks T., Willey R., Rabson A., Martin M. (1986). Production of acquired immunodeficiency syndrome-associated retrovirus in human and nonhuman cells transfected with an infectious molecular clone. J. Virol..

[66] Zhu H., Jian H., Zhao L.-J. (2004). Identification of the 15FRFG domain in HIV-1 Gag p6 essential for Vpr packaging into the virion. Retrovirology.

[67] Kapust R., Tözsér J., Fox J., Anderson D., Cherry S., Copeland T., Waugh D. (2001). Tobacco etch virus protease: mechanism of autolysis and rational design of stable mutants with wild-type catalytic proficiency. Protein Eng..

[68] Tropea J., Cherry S., Waugh D. (2009). Expression and purification of soluble His(6)-tagged TEV protease. Methods Mol. Biol..

[69] Lee B., De Guzman R., Turner B., Tjandra N., Summers M. (1998). Dynamical behavior of the HIV-1 nucleocapsid protein. J. Mol. Biol..

[70] Hargittai M., Musier-Forsyth K. (2000). Use of terbium as a probe of tRNA tertiary structure and folding. RNA.

[71] Hargittai M., Mangla A., Gorelick R.J., Musier-Forsyth K. (2001). HIV-1 nucleocapsid protein zinc finger structures induce tRNA(Lys,3) structural changes but are not critical for primer/template annealing. J. Mol. Biol..

[72] Milligan J., Groebe D., Witherell G., Uhlenbeck O. (1987). Oligoribonucleotide synthesis using T7 RNA polymerase and synthetic DNA templates. Nucleic Acids Res..

[73] Pagano J., Farley B., McCoig L., Ryder S. (2007). Molecular basis of RNA recognition by the embryonic polarity determinant MEX-5. J. Biol. Chem..

[74] Jones C., Saadatmand J., Kleiman L., Musier-Forsyth K. (2013). Molecular mimicry of human tRNA^Lys^ anti-codon domain by HIV-1 RNA genome facilitates tRNA primer annealing. RNA.

[75] Stewart-Maynard K., Cruceanu M., Wang F., Vo M.-N., Gorelick R.J., Williams M.C., Rouzina I., Musier-Forsyth K. (2008). Retroviral nucleocapsid proteins display nonequivalent levels of nucleic acid chaperone activity. J. Virol..

[76] Webb J., Jones C., Parent L., Rouzina I., Musier-Forsyth K. (2013). Distinct binding interactions of HIV-1 Gag to Psi and non-Psi RNAs: implications for viral genomic RNA packaging. RNA.

[77] Hargittai M., Gorelick R.J., Rouzina I., Musier-Forsyth K. (2004). Mechanistic insights into the kinetics of HIV-1 nucleocapsid protein-facilitated tRNA annealing to the primer binding site. J. Mol. Biol..

[78] Bernacchi S., Stoylov S., Piémont E., Ficheux D., Roques B., Darlix J.-L., Mély Y. (2002). HIV-1 nucleocapsid protein activates transient melting of least stable parts of the secondary structure of TAR and its complementary sequence. J. Mol. Biol..

[79] Grzesiek S., Döbeli H., Gentz R., Garotta G., Labhardt A., Bax A. (1992). 1H, 13C, and 15N NMR backbone assignments and secondary structure of human interferon-gamma. Biochemistry.

[80] Delaglio F., Grzesiek S., Vuister G., Zhu G., Pfeifer J., Bax A. (1995). NMRPipe: a multidimensional spectral processing system based on UNIX pipes. J. Biomol. NMR.

[81] Johnson B., Blevins R. (1994). NMR View: a computer program for the visualization and analysis of NMR data. J. Biomol. NMR.

[82] McCauley M.J., Williams M.C. (2009). Optical tweezers experiments resolve distinct modes of DNA-protein binding. Biopolymers.

[83] Morcock D., Thomas J., Sowder R., Henderson L., Crise B., Gorelick R.J. (2008). HIV-1 inactivation by 4-vinylpyridine is enhanced by dissociating Zn(2+) from nucleocapsid protein. Virology.

[84] Cruceanu M., Gorelick R.J., Musier-Forsyth K., Rouzina I., Williams M.C. (2006). Rapid kinetics of protein-nucleic acid interaction is a major component of HIV-1 nucleocapsid protein's nucleic acid chaperone function. J. Mol. Biol..

[85] Kovaleski B., Kennedy R., Khorchid A., Kleiman L., Matsuo H., Musier-Forsyth K. (2007). Critical role of helix 4 of HIV-1 capsid C-terminal domain in interactions with human lysyl-tRNA synthetase. J. Biol. Chem..

[86] Solbak S., Reksten T., Hahn F., Wray V., Henklein P., Henklein P., Halskau Ø., Schubert U., Fossen T. (2013). HIV-1 p6 - a structured to flexible multifunctional membrane-interacting protein. Biochim. Biophys. Acta.

[87] Morellet N., de Rocquigny H., Mély Y., Jullian N., Déméné H., Ottmann M., Gérard D., Darlix J.-L., Fournie-Zaluski M., Roques B. (1994). Conformational behaviour of the active and inactive forms of the nucleocapsid NCp7 of HIV-1 studied by 1H NMR. J. Mol. Biol..

[88] Paramanathan T., Vladescu I., McCauley M.J., Rouzina I., Williams M.C. (2012). Force spectroscopy reveals the DNA structural dynamics that govern the slow binding of Actinomycin D. Nucleic Acids Res..

[89] Wu H., Mitra M., McCauley M.J., Thomas J.A., Rouzina I., Musier-Forsyth K., Williams M.C., Gorelick R.J. (2013). Aromatic residue mutations reveal direct correlation between HIV-1 nucleocapsid protein's nucleic acid chaperone activity and retroviral replication. Virus Res..

[90] Vladescu I.D., McCauley M.J., Nunez M.E., Rouzina I., Williams M.C. (2007). Quantifying force-dependent and zero-force DNA intercalation by single-molecule stretching. Nat. Methods.

[91] Vladescu I.D., McCauley M.J., Rouzina I., Williams M.C. (2005). Mapping the phase diagram of single DNA molecule force-induced melting in the presence of ethidium. Phys. Rev. Lett..

[92] Paramanathan T., Westerlund F., McCauley M.J., Rouzina I., Lincoln P., Williams M.C. (2008). Mechanically manipulating the DNA threading intercalation rate. J. Am. Chem. Soc..

[93] Kim S.-E., Liu F., Im Y., Stephen A., Fivash M., Waheed A., Freed E., Fisher R., Hurley J., Burke T. (2011). Elucidation of new binding interactions with the tumor susceptibility gene 101 (Tsg101) protein using modified HIV-1 Gag-p6 derived peptide ligands. ACS Med. Chem. Lett..

[94] Kaplan A.H., Manchester M., Swanstrom R. (1994). The activity of the protease of human immunodeficiency virus type 1 is initiated at the membrane of infected cells before the release of viral proteins and is required for release to occur with maximum efficiency. J. Virol..

[95] Jalalirad M., Laughrea M. (2010). Formation of immature and mature genomic RNA dimers in wild-type and protease-inactive HIV-1: differential roles of the Gag polyprotein, nucleocapsid proteins NCp15, NCp9, NCp7, and the dimerization initiation site. Virology.

[96] Mirambeau G., Lyonnais S., Coulaud D., Hameau L., Lafosse S., Jeusset J., Justome A., Delain E., Gorelick R.J., Le Cam E. (2006). Transmission electron microscopy reveals an optimal HIV-1 nucleocapsid aggregation with single-stranded nucleic acids and the mature HIV-1 nucleocapsid protein. J. Mol. Biol..

[97] Mirambeau G., Lyonnais S., Coulaud D., Hameau L., Lafosse S., Jeusset J., Borde I., Reboud-Ravaux M., Restle T., Gorelick R.J. (2007). HIV-1 protease and reverse transcriptase control the architecture of their nucleocapsid partner. PloS ONE.

[98] Rouzina I., Bloomfield V.A. (1996). Macroion attraction due to electrostatic correlation between screening counterions .1. Mobile surface-adsorbed ions and diffuse ion cloud. J. Phys. Chem..

[99] Nguyen T.T., Rouzina I., Shklovskii B.I. (2000). Reentrant condensation of DNA induced by multivalent counterions. J. Chem. Phys..

[100] Pelta J., Livolant F., Sikorav J.L. (1996). DNA condensation by polyamines and cobalthexamine. J. Biol. Chem..

[101] Bloomfield V.A. (1997). DNA condensation by multivalent cations. Biopolymers.

[102] Stoylov S., Vuilleumier C., Stoylova E., De Rocquigny H., Roques B., Gérard D., Mély Y. (1997). Ordered aggregation of ribonucleic acids by the human immunodeficiency virus type 1 nucleocapsid protein. Biopolymers.

[103] Le Cam E., Coulaud D., Delain E., Petitjean P., Roques B., Gérard D., Stoylova E., Vuilleumier C., Stoylov S., Mély Y. (1998). Properties and growth mechanism of the ordered aggregation of a model RNA by the HIV-1 nucleocapsid protein: an electron microscopy investigation. Biopolymers.

[104] Sheng N., Erickson-Viitanen S. (1994). Cleavage of p15 protein in vitro by human immunodeficiency virus type 1 protease is RNA dependent. J. Virol..

[105] Sheng N., Pettit S., Tritch R., Ozturk D., Rayner M., Swanstrom R., Erickson-Viitanen S. (1997). Determinants of the human immunodeficiency virus type 1 p15NC-RNA interaction that affect enhanced cleavage by the viral protease. J. Virol..

[106] Rouzina I., Bloomfield V.A. (1996). Influence of ligand spatial organization on competitive electrostatic binding to DNA. J. Phys. Chem..

[107] Rouzina I., Bloomfield V. (1997). Competitive electrostatic binding of charged ligands to polyelectrolytes: practical approach using the non-linear Poisson-Boltzmann equation. Biophys. Chem..

[108] Pant K., Karpel R.L., Rouzina I., Williams M.C. (2004). Mechanical measurement of single-molecule binding rates: kinetics of DNA helix-destabilization by T4 gene 32 protein. J. Mol. Biol..

[109] Pant K., Karpel R., Rouzina I., Williams M.C. (2005). Salt dependent binding of T4 gene 32 protein to single and double-stranded DNA: single molecule force spectroscopy measurements. J. Mol. Biol..

[110] Rouzina I., Pant K., Karpel R., Williams M.C. (2005). Theory of electrostatically regulated binding of T4 gene 32 protein to single- and double-stranded DNA. Biophys. J..

[111] Kowalczykowski S., Lonberg N., Newport J., Paul L., von Hippel P. (1980). On the thermodynamics and kinetics of the cooperative binding of bacteriophage T4-coded gene 32 (helix destabilizing) protein to nucleic acid lattices. Biophys. J..

[112] Villemain J.L., Giedroc D.P. (1996). The N-terminal B-domain of T4 gene 32 protein modulates the lifetime of cooperatively bound Gp32−ss nucleic acid complexes. Biochemistry.

[113] Waidner L., Flynn E., Wu M., Li X., Karpel R. (2001). Domain effects on the DNA-interactive properties of bacteriophage T4 gene 32 protein. J. Biol. Chem..

[114] Pant K., Shokri L., Karpel R., Morrical S., Williams M.C. (2008). Modulation of T4 gene 32 protein DNA binding activity by the recombination mediator protein UvsY. J. Mol. Biol..

[115] Kaplan A., Zack J., Knigge M., Paul D., Kempf D., Norbeck D., Swanstrom R. (1993). Partial inhibition of the human immunodeficiency virus type 1 protease results in aberrant virus assembly and the formation of noninfectious particles. J. Virol..

[116] Müller B., Anders M., Akiyama H., Welsch S., Glass B., Nikovics K., Clavel F., Tervo H.-M., Keppler O., Kräusslich H.-G. (2009). HIV-1 Gag processing intermediates trans-dominantly interfere with HIV-1 infectivity. J. Biol. Chem..

[117] Keller P., Adamson C., Heymann J., Freed E., Steven A. (2011). HIV-1 maturation inhibitor bevirimat stabilizes the immature Gag lattice. J. Virol..

[118] Yang Y., Fricke T., Diaz-Griffero F. (2013). Inhibition of reverse transcriptase activity increases stability of the HIV-1 core. J. Virol..

[119] Forshey B., von Schwedler U., Sundquist W., Aiken C. (2002). Formation of a human immunodeficiency virus type 1 core of optimal stability is crucial for viral replication. J. Virol..

[120] Hulme A., Perez O., Hope T. (2011). Complementary assays reveal a relationship between HIV-1 uncoating and reverse transcription. Proc. Natl. Acad. Sci. U.S.A..

