# Supplementary material for: Distinct nucleic acid interaction properties of HIV-1 nucleocapsid protein precursor NCp15 explain reduced viral infectivity
Source: Nucleic Acids Res. 2014 May 9;42(11):7145–59. doi: 10.1093/nar/gku335 (PMC4066767; doi:10.1093/nar/gku335)
Supplement: SUPPLEMENTARY DATA [file supp_42_11_7145__index.html]

SUPPLEMENTARY DATA 

# Distinct nucleic acid interaction properties of HIV-1 nucleocapsid protein precursor NCp15 explain reduced viral infectivity

## SUPPLEMENTARY DATA

**Files in this Data Supplement:**

- Supplementary Data
