# Supplementary material for: Distinct nucleic acid interaction properties of HIV-1 nucleocapsid protein precursor NCp15 explain reduced viral infectivity
Source: Nucleic Acids Res. 2014 May 9;42(11):7145–59. doi: 10.1093/nar/gku335 (PMC4066767; doi:10.1093/nar/gku335)
Supplement: SUPPLEMENTARY DATA [file supp_gku335_nar-00038-v-2014-File018.pdf]

Supplementary Data

For the manuscript "Distinct nucleic acid interaction properties of HIV-1 nucleocapsid protein precursor NCp15 explain reduced viral infectivity", by Wei Wang, Nada Naiyer, Mithun Mitra, Jialin Li**,** Mark C. Williams, Ioulia Rouzina, Robert J. Gorelick, Zhengrong Wu, and Karin Musier-Forsyth

Figure S1	Annealing of $tRNA^{Lys3}$ by HIV-1 NCp7 proteins prepared by different methods.
Figure S2	Representative gel-shift annealing assays performed with HIV-1 NC variants.
Figure S3	Immunoblot of recombinant NC proteins, and rapid harvest (30 min) and 46 h HIV-1 from transfected 293 cells.
Figure S4	Annealing of $tRNA^{Lys3}$ by combinations of NCp9 and NCp7/NCp15.
Figure S5	The relative populations of the four states of cTAR DNA as determined by time-resolved FRET.
Figure S6	The size distribution of RNA alone and NCp15 alone determined by DLS.
Figure S7	Time-course annealing reactions of NCp9 and NCp15 at different salt concentrations.
Figure S8	Stretching curves of HIV-1 NCp7, NCp9 and NCp15 under different pulling rates.
Figure S9	Immunoblot analysis shows that an additional protease cleavage site was introduced upon mutating the 8 D/E to A in the context of NCp15.

Figure S1

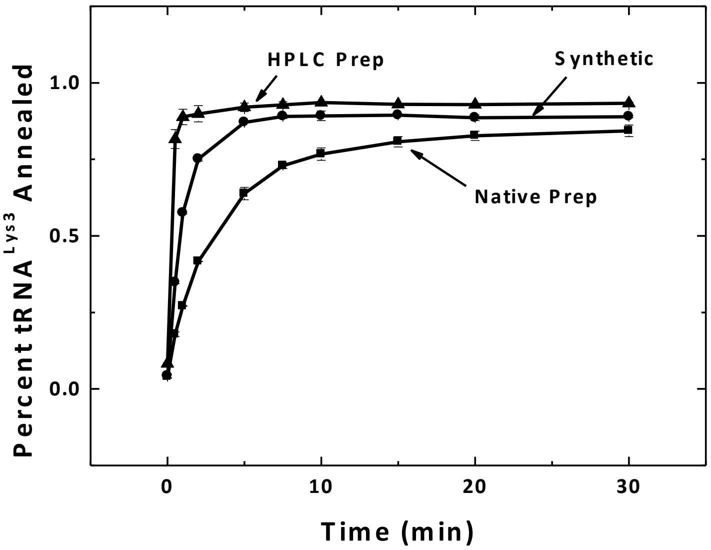

Figure S1. HIV-1 NCp7 proteins (2 μM), prepared by different methods as described in the main text, are all capable of annealing $tRNA^{Lys3}$ to the ShortPBS.

Figure S2

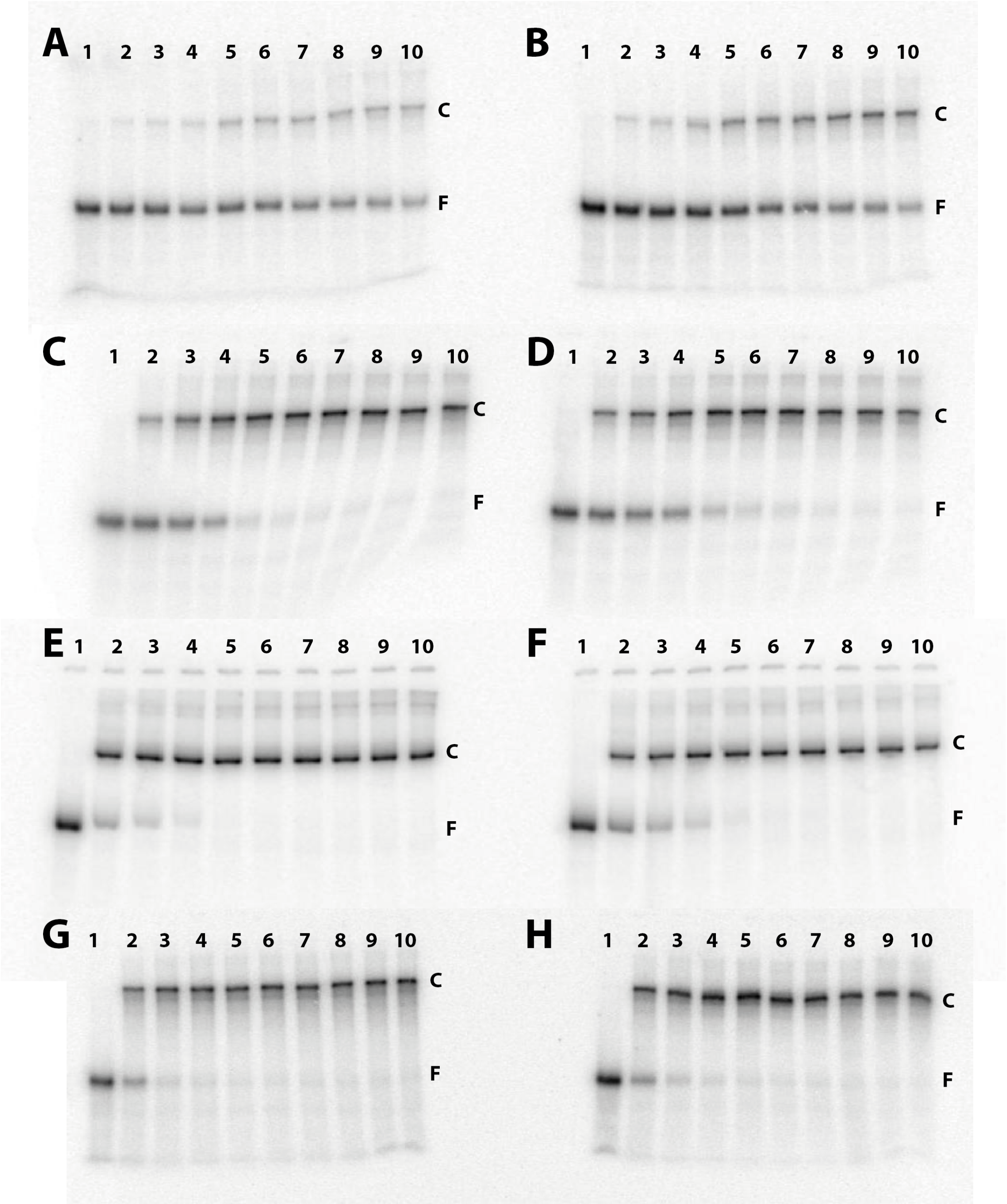

Figure S2. Representative gel-shift annealing assays performed with HIV-1 NC variants. (A) NCp7 at 600 nM, (B) NCp15 at 600 nM, (C) NCp7 at 2 μM, (D) NCp15 at 2 μM, (E) NCp9 at 600 nM, (F) NCp15-8A at 600 nM, (G) NCp15-5A at 600 nM and (H) NCp15 C3A at 600 nM. In all cases lanes 1-10 are time points taken at 0, 0.5, 1, 2, 5, 7.5, 10, 15, 20, 30 min. The free [^{32}P]-tRNA band is labeled F and the tRNA/ShortPBS complex band is labeled C.

Figure S3

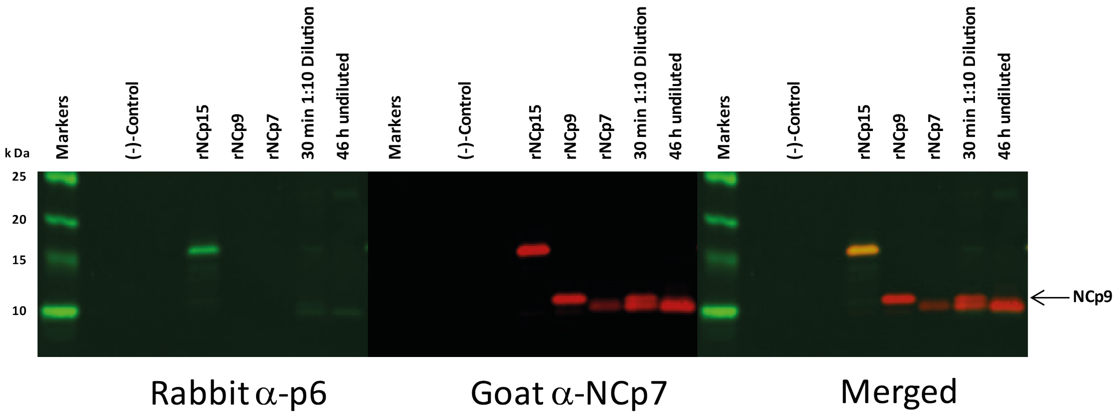

Figure S3. Immunoblot of recombinant NC proteins (rNCp7, rNCp9, and rNCp15) and 30 min and 46 h harvested HIV-1 from transfected 293T cells. The immunoblot of p6-containing proteins using rabbit α-p6 serum is shown in the left panel, NCp7-containing proteins using goat α-NCp7 serum are shown in the middle panel and the merged image is shown on the right. Marker positions are indicated on the left. Wild-type virus was isolated as described previously (121) using Mirus Bio's (Madison WI) TransIT 293 reagent according to the manufacturer's instructions. After 24 h, flasks were fluid changed and incubated for either an additional 30 min or 46 h. The 30 min viral sups were maintained on ice in the presence of 20 μM Saquinavir to inhibit viral protease activity (122). The culture fluids were passed through 0.22 μm Millex-GS filters and centrifuged at 140,000 ×g, 4 °C for 2 h as described (121). Viral pellets from each sample were subjected to sodium dodecyl sulfate-polyacrylamide gel electrophoresis fractionation on Novex 4-20% Tris-Glycine precast gels (Life Technologies, Grand Island, NY). Immunoblotting was performed essentially as described in Gorelick et al. (121) except that near-infrared detection was performed using LI-COR Biosciences (Lincoln, Nebraska) reagents as recommended by the manufacturer. Primary goat anti-NCp7 (AIDS and Cancer Virus Program, #77, 001155) and rabbit anti-p6 sera (AIDS and Cancer Virus Program, DJ-30552, 0072692) were used at 1:5000 and 1:20,000 dilutions, respectively. IRDye 680LT donkey anti-goat and IRDye 800CW donkey anti-rabbit secondary antibodies were used at dilutions of 1:10,000. The Odyssey Imaging System was used to detect protein bands (LI-COR Biosciences).

Figure S4

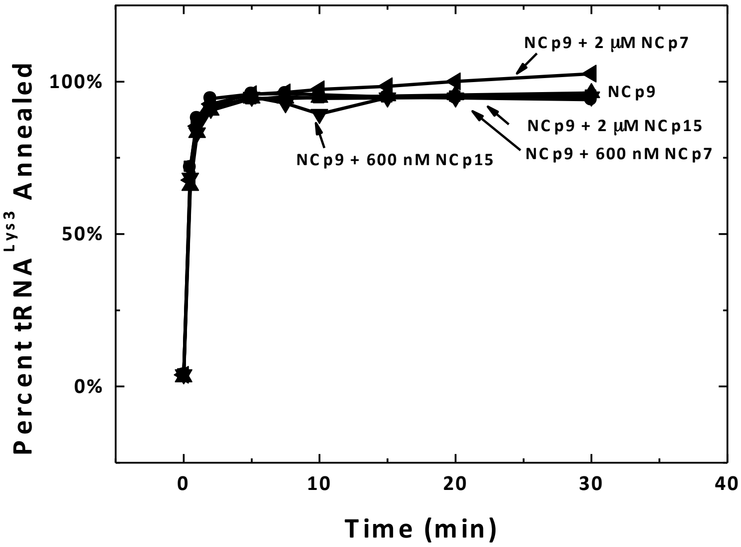

Figure S4. Annealing of $tRNA^{Lys3}$ to ShortPBS in the presence of combinations of different NC forms. In these experiments, 600 nM NCp9 was preincubated with NCp7 (600 nM or 2 μM) or NCp15 (600 nM or 2 μM) and the reaction was initiated with the mixture. An assay with NCp9 alone (600 nM) is shown for comparison.

Figure S5

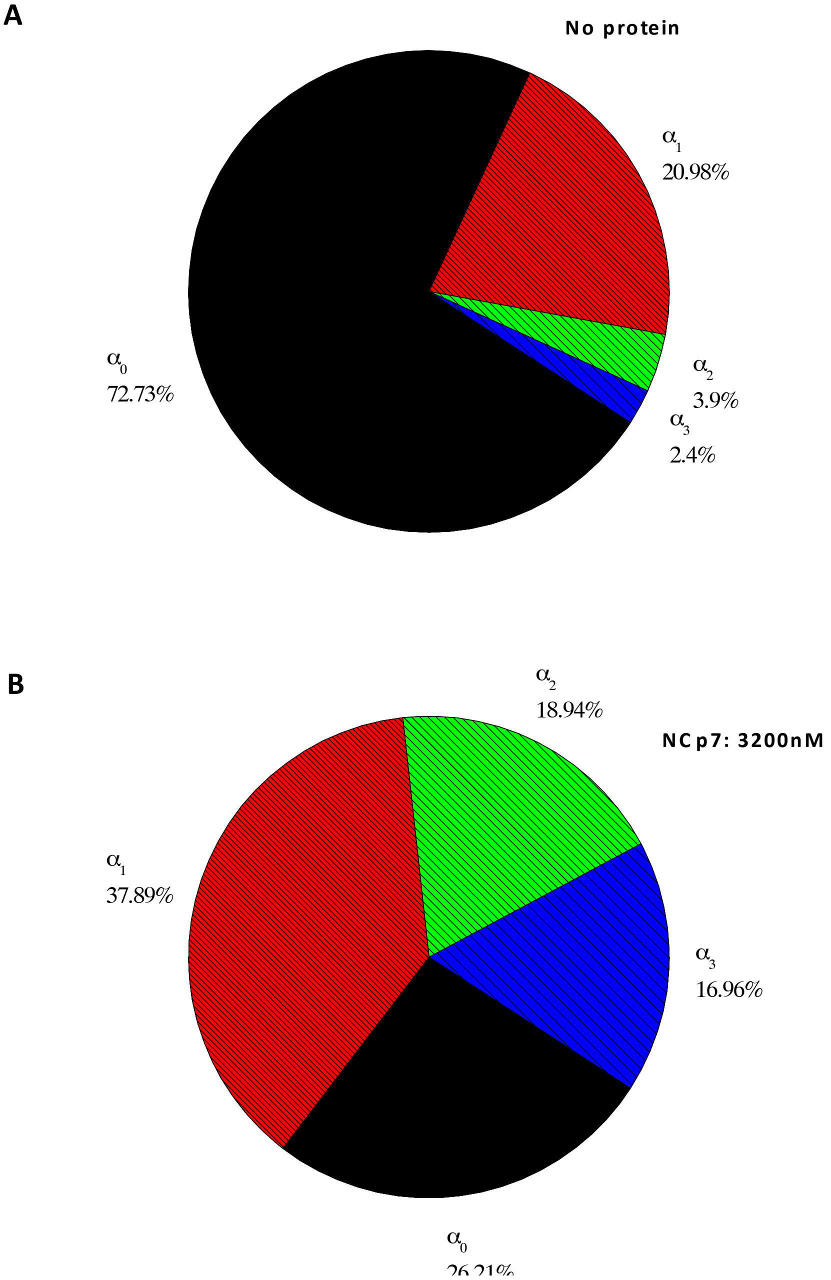

[illegible]

Continued

Figure S5 Continued

C

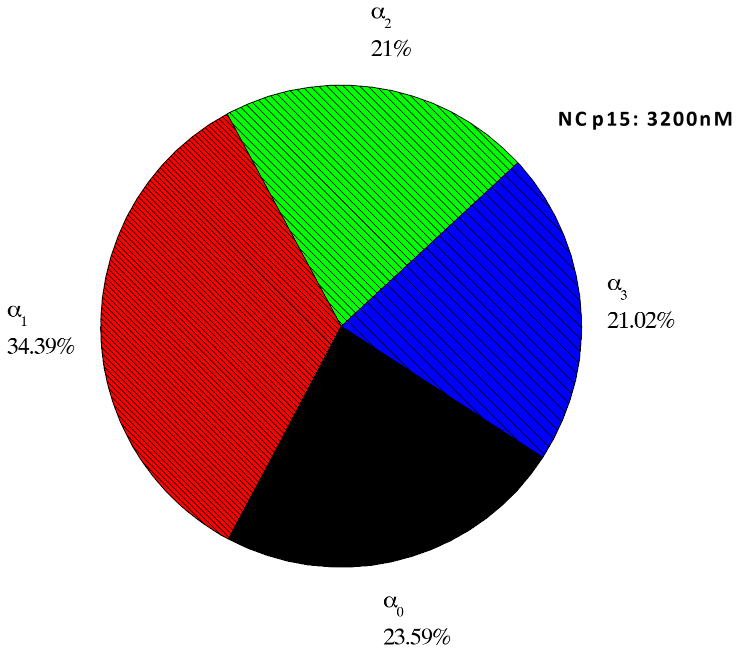

D

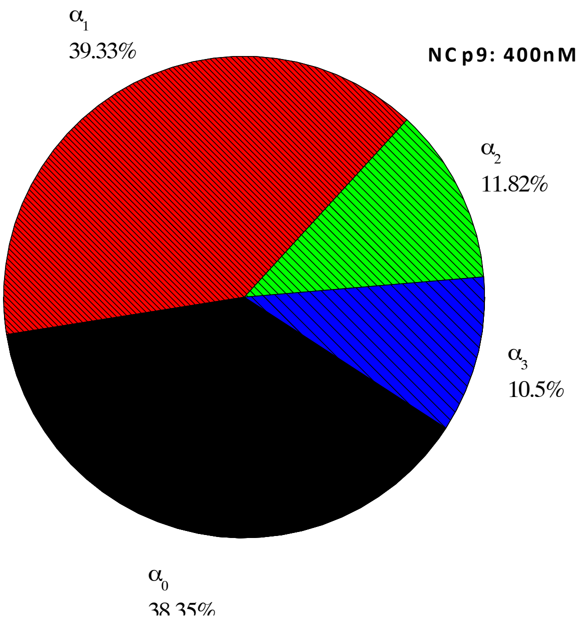

Figure S5. The relative populations of the four states of cTAR DNA as determined by time-resolved FRET. (A) In the absence of NC the dark (closed) state (α_0) is dominant. In the presence of saturating NCp7 (B) or NCp15 (C), more equal populations of all states are observed, showing that both proteins display similar levels of nucleic acid destabilizing activity. Due to nucleic acid aggregation at higher concentrations, only 400 nM NCp9 could be used. Under these conditions, NCp9 still demonstrate significant nucleic acid destabilization capabilities (D).

Figure S6

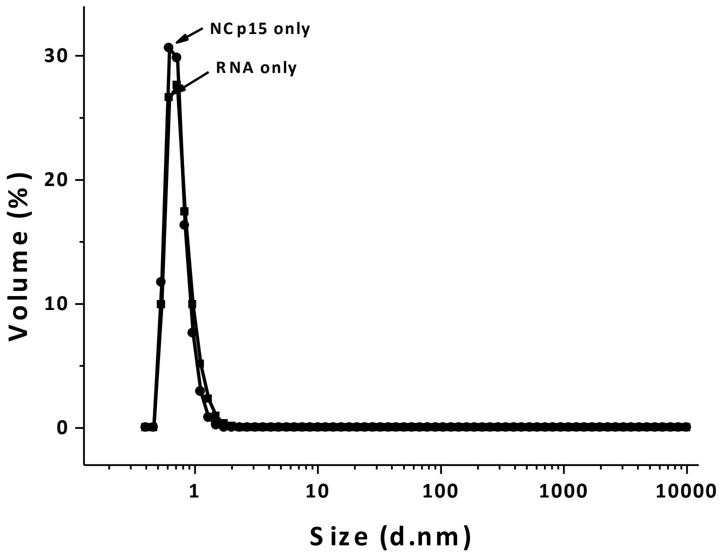

Figure S6. The size distribution of RNA and NCp15 determined by DLS. The RNA and NCp15 concentrations are the same as in mixed reactions.

Figure S7

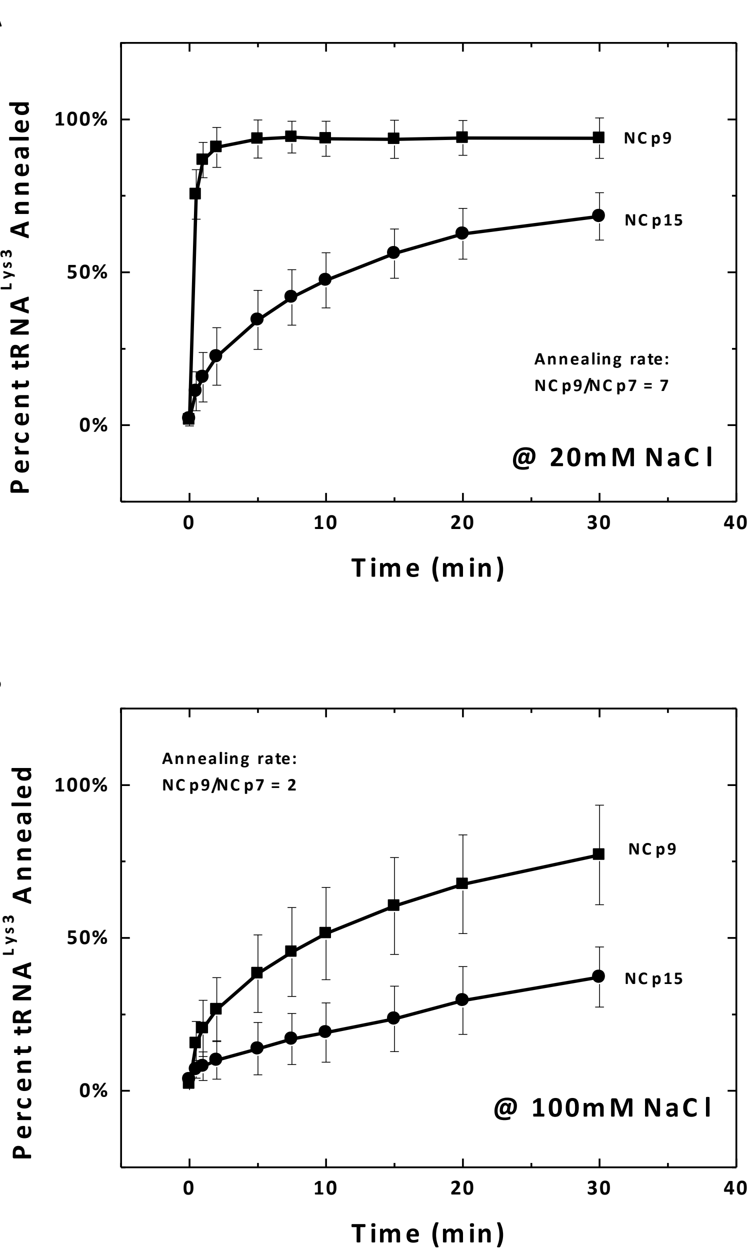

Continued

Figure S7 Continued

C

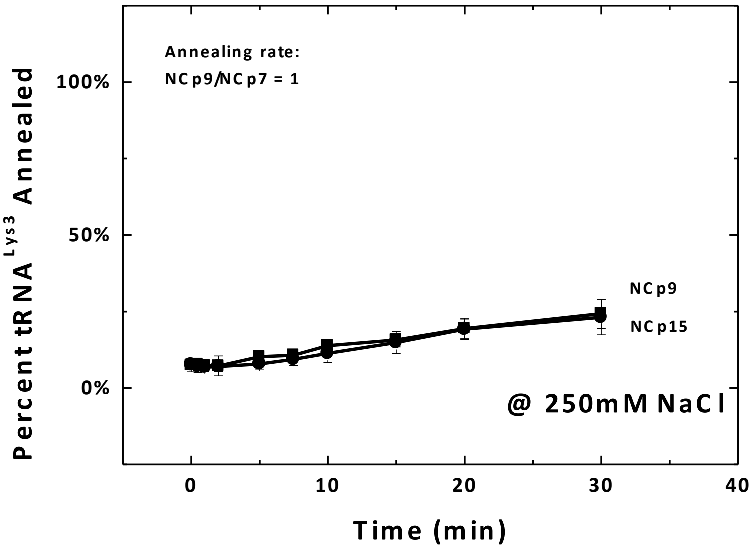

Figure S7. Time-course annealing reactions of NCp9 and NCp15 at (A) 20 mM, (B) 100 mM and (C) 250 mM NaCl. At higher concentrations of NaCl, the annealing rate of NCp15 approaches that of NCp9 under the same conditions. The annealing rates were determined by fitting the data to a single-exponential equation.

Figure S8

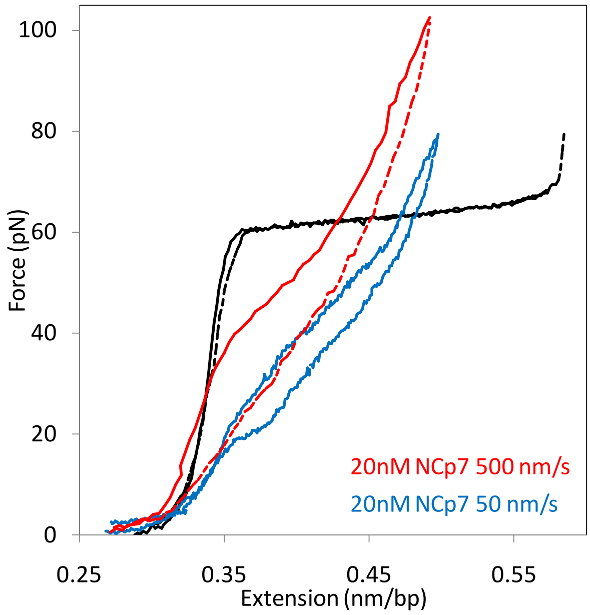

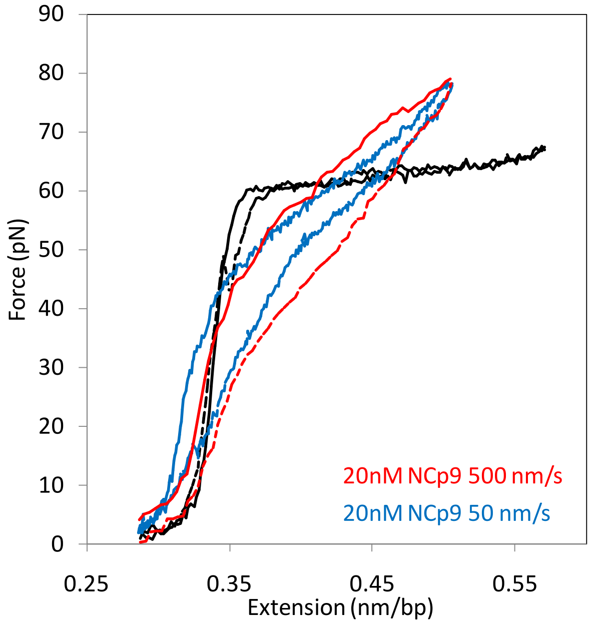

Continued

Figure S8 Continued

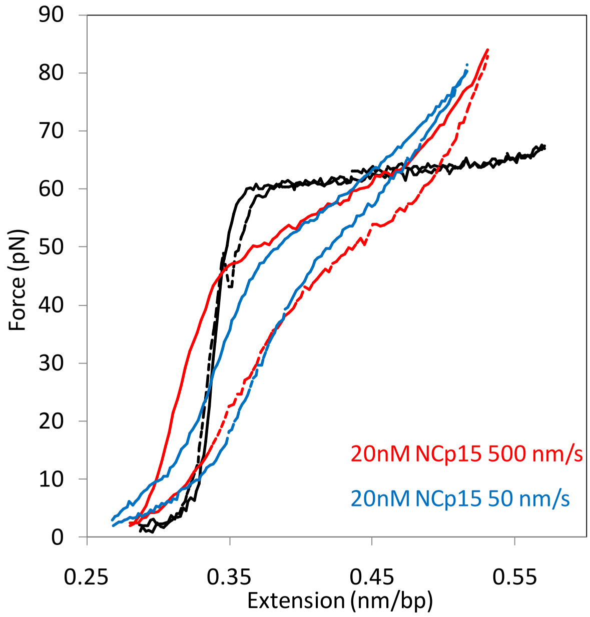

Figure S8. Stretching curves of HIV-1 (A) NCp7, (B) NCp9 and (C) NCp15 under different pulling rates. For all the 3 proteins, hysteresis increases when the pulling rate is faster.

Figure S9

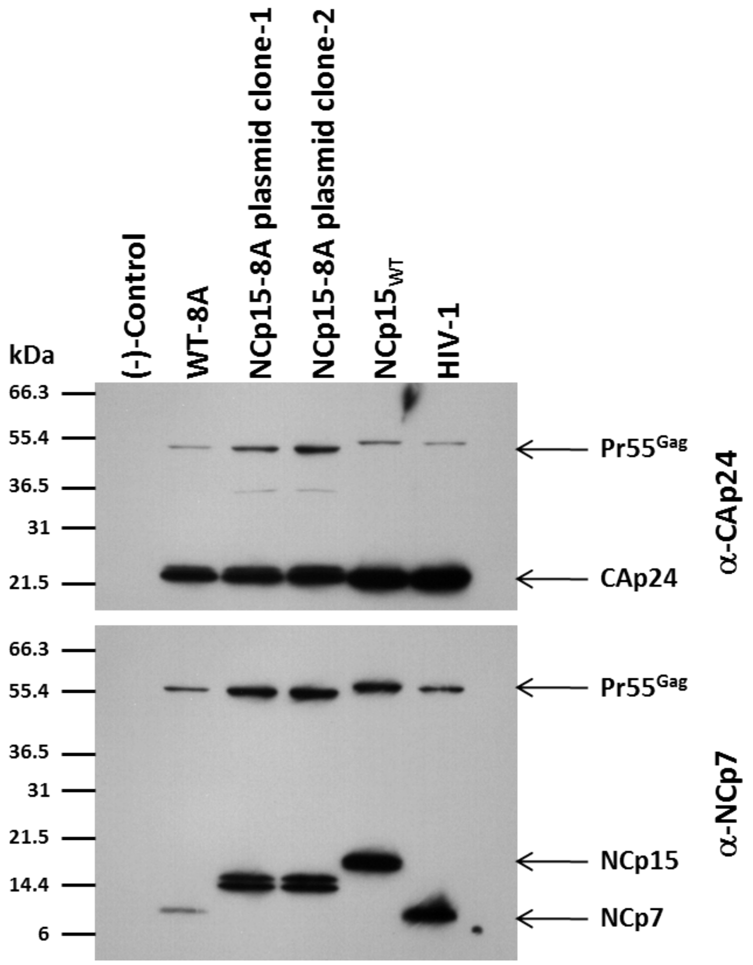

Figure S9. Immunoblot analysis shows that an additional protease cleavage site was introduced upon mutating the 8 D/E to A in the context of NCp15. Viruses were derived by transfection as described in Materials and Methods. Virions were collected by ultracentrifugation at 235,000×*g* for 1 h at 4 °C through a 3 mL 20% (wt./vol.) sucrose cushion (in phosphate buffered saline) using a Beckman SW-41Ti rotor (Beckman-Coulter, Inc., Brea, CA). Semidry immunoblot analysis was performed as previously described (123). Proteins from virions, normalized for equivalent exogenous RT activities were detected by sequential incubation of the blot with primary goat polyclonal antisera against CAp24 (goat 81) or NCp7 (goat 77) from the AIDS and Cancer Virus Program, Frederick National Laboratory for Cancer Research, Frederick, MD. Blot images were obtained using horseradish peroxidase-conjugated anti-goat secondary antibody (Biochain Institute, Hayward, CA) and an Immun-Star Horseradish Peroxidase Substrate Kit (Bio-Rad, Hercules, CA) with LumiFilm (Roche Applied Science, Indianapolis, IN). Molecular weight markers are shown on the left and positions of proteins and antibodies used are shown on the right.

Table S1. Kinetic parameters describing the non-equilibrium DNA-binding component of NC molecules, *F(x)*.

Protein, 20 nM	f_{fast}	τ_{fast} , s	f_{slow}	τ_{slow} , s
NCp7	0.14±0.05	35±15	0.22±0.02	1,200±500
NCp9	0.15±0.05	45±15	0.23±0.02	5,220±1000
NCp15	0.19±0.05	40±15	0.30±0.02	1,370±1000

Parameters in this Table were obtained by fitting the measured non-equilibrium component of DNA-bound NC presented in Fig. 7 to Eq. 7.

References

121. Gorelick, R.J., Nigida, S.M., Jr., Bess, J.W., Jr., Arthur, L.O., Henderson, L.E. and Rein, A. (1990) Noninfectious human immunodeficiency virus type 1 mutants deficient in genomic RNA. *J. Virol.*, **64**, 3207-3211.

122. Ott, D.E., Coren, L.V. and Shatzer, T. (2009) The nucleocapsid region of human immunodeficiency virus type 1 Gag assists in the coordination of assembly and Gag processing: role for RNA-Gag binding in the early stages of assembly. *J. Virol.*, **83**, 7718-7727.

123. Ott, D.E., Coren, L.V., Gagliardi, T.D. and Nagashima, K. (2005) Heterologous Late-Domain Sequences Have Various Abilities To Promote Budding of Human Immunodeficiency Virus Type 1. *J. Virol.*, **79**, 9038-9045.
